# Eye Movement Desensitization and Reprocessing Therapy in Persons With Personality Disorders: A Randomized Clinical Trial

**DOI:** 10.1001/jamanetworkopen.2025.33421

**Published:** 2025-09-25

**Authors:** Simon Hofman, Laurian Hafkemeijer, Ad de Jongh, Cristina W. Slotema

**Affiliations:** 1 Department of Personality Disorders, Parnassia Psychiatric Institute, the Hague, the Netherlands; 2 Department of Psychology, Education and Child Studies, Erasmus School of Social and Behavioral Sciences, Erasmus University Rotterdam, Rotterdam, the Netherlands; 3 GGZ Delfland, Delft, the Netherlands; 4 Academic Centre for Dentistry Amsterdam, University of Amsterdam and VU University, Amsterdam, the Netherlands; 5 Research Department, PSYTREC (Psychotrauma Expertise Center), Bilthoven, the Netherlands; 6 School of Health Sciences, Salford University, Manchester, United Kingdom; 7 Institute of Health and Society, University of Worcester, Worcester, United Kingdom; 8 School of Psychology, Queen’s University, Belfast, Ireland

## Abstract

**Importance:**

Adverse childhood experiences contribute to the development of personality disorders (PDs). Although trauma-focused interventions are effective for posttraumatic stress disorder (PTSD), their effect on PD symptoms is less established.

**Objective:**

To evaluate the effectiveness of eye movement desensitization and reprocessing (EMDR) therapy in reducing PD symptoms compared with a waiting list, regardless of PTSD status.

**Design, Setting, and Participants:**

This 2-arm, multicenter, single-blind, randomized clinical trial was performed in the specialized outpatient departments of 2 clinics in the Netherlands from February 22, 2021, to October 2, 2024. Participants included 159 patients with PD diagnosed using the Structured Clinical Interview for *DSM-5* Personality Disorders (SCID-5-PD). Data were analyzed based on intention to treat.

**Intervention:**

Ten 90-minute EMDR sessions for 5 weeks, targeting traumatic and adverse memories linked to PD symptoms.

**Main Outcomes and Measures:**

Pretreatment, posttreatment, and 3-month follow-up assessments using the Assessment of *DSM-IV* Personality Disorders (ADP-IV), SCID-5-PD, Level of Personality Functioning Scale (LPFS), and Difficulties in Emotion Regulation Scale (DERS).

**Results:**

Among the 159 patients included in the analysis, mean (SD) age was 35.4 (12.0) years, and 130 were female (81.8%). Seventy-nine participants were randomized to the EMDR group and 80 to the waiting-list control group. Four participants (5.1%) dropped out of the EMDR group, and 16 (20.3%) were early completers, without adverse events. EMDR therapy outperformed the waiting-list condition for ADP-IV post treatment (β, −37.93 [95% CI, −52.54 to −23.33]; *P* < .001; Cohen *d* = 0.31 [95% CI, −0.05 to 0.66]) and at follow-up (β, −45.73 [95% CI, −64.90 to −26.56]; *P* < .001; Cohen *d* = 0.46 [95% CI, 0.10-0.82]), SCID-5-PD post treatment (β, −3.65 [95% CI, −5.87 to −1.42]; *P* = .002; *d* = 0.48 [95% CI, 0.14-0.82]) and at follow-up (β, −3.70 [95% CI, −7.10 to −0.30]; *P* = .03; Cohen *d* = 0.61 [95% CI, 0.25-0.97]), LPFS post treatment (β, −3.13 [95% CI, −4.86 to −1.41]; *P* < .001; Cohen *d* = 0.31 [95% CI, −0.05 to 0.67]) and at follow-up (β, −3.62 [95% CI, −5.96 to −1.28]; *P* = .003; Cohen *d* = 0.43 [95% CI, 0.06-0.79]), and DERS post treatment (β, −9.03 [95% CI, −14.90 to −3.15]; *P* = .003; Cohen *d* = 0.35 [95% CI, −0.01 to 0.71]) and at follow-up (β, −11.73 [95% CI, −19.90 to −3.55]; *P* = .005; Cohen *d* = 0.62 [95% CI, 0.25-0.98]). PD remission was more common in the EMDR than control groups both post treatment (ADP-IV, 38.3% vs 6.8%; SCID-5-PD, 33.3% vs 7.8%) and at follow-up (ADP-IV, 45.4% vs 5.9%; SCID-5-PD, 44.1% vs 15.8%).

**Conclusions and Relevance:**

In this randomized clinical trial of 159 patients with PD, EMDR therapy led to significant reduction in PD symptoms, with 30 (44.1%) achieving remission. These findings support the potential of EMDR therapy for PD treatment and encourage further confirmatory research.

**Trial Registration:**

Netherlands Trial Register: NL9078

## Key Points

**Question:**

Does eye movement desensitization and reprocessing (EMDR) therapy reduce personality disorder (PD) symptoms, regardless of posttraumatic stress disorder status?

**Findings:**

In this randomized clinical trial including 159 patients with PD, results of EMDR therapy were superior to those of a waiting-list control group in reducing PD symptoms post treatment and at follow-up. PD remission was significantly more common in the EMDR group compared with the control group at both time points.

**Meaning:**

In this study, EMDR therapy demonstrated clinically meaningful reductions in PD symptoms, with nearly one-half of participants achieving diagnostic remission, supporting its potential as an effective intervention for PDs.

## Teaser

This multicenter randomized clinical trial investigates the effectiveness of eye movement desensitization and reprocessing therapy for patients with personality disorders, with and without comorbid posttraumatic stress disorder.

## Introduction

Personality disorders (PDs) are a substantial public health concern, affecting approximately 12% of the general population^1^ and as many as 50% of individuals in psychiatric outpatient settings.^2^ PDs cause considerable problems for individuals and affect families and communities, contributing to substantial societal costs.^3,4^ Although psychotherapy is an effective first-line treatment, it typically requires long-term commitment, often exceeding 1 year, at least for individuals with clinical or subclinical borderline PD.^5^

Given the association between adverse childhood experiences and the development of PDs,^6,7^ trauma-focused therapy has gained attention as a potential treatment for PDs.^8^ Research indicates that trauma-focused therapies significantly reduce PTSD symptoms in patients with comorbid borderline PD and PTSD (Hedges *g* range, 1.04-1.31), with minimal adverse events.^9,10^ Moreover, treating PTSD in patients with a comorbid PD appears to be safe and effective (Hedges *g* = 0.52),^9^ with adverse events reported rarely and of minimal severity. ^11,12,13,14,15^

Eye movement desensitization and reprocessing (EMDR) therapy is a well-established treatment for the processing of traumatic memories^16,17,18^ and has demonstrated promise for reducing psychological distress and improving functioning in individuals with PDs, even in the absence of comorbid PTSD.^19^ A randomized clinical trial in patients with PD without comorbid PTSD^8,19,20,21^ demonstrated that 5 EMDR sessions targeting adverse childhood experiences such as emotional abuse or neglect led to moderate reductions on the Brief Symptom Inventory (Cohen *d* = 0.65) and improvements in personality functioning (Cohen *d* = 0.56), without adverse events and less symptom exacerbation than in the waiting-list control group. This study did not assess PD symptom severity, diagnostic remission, or severity of dysregulation of emotions and administered only 5 weekly EMDR sessions.

To address these gaps and advance the development of a brief and effective therapeutic approach for PDs, the present study investigated the effectiveness of EMDR therapy for PDs, regardless of the presence of PTSD. The primary aim was to determine whether EMDR therapy could significantly reduce PD symptoms compared with a waiting-list control condition. We hypothesized that EMDR therapy would lead to a significant reduction in PD symptom severity compared with the control condition. Secondary objectives included evaluating diagnostic remission, changes in personality functioning, and emotion regulation difficulties.

## Methods

### Design

This study was part of a multicenter single-blind superiority randomized clinical with 2 arms, comparing EMDR therapy and a waiting-list control condition (1:1) (the Trauma-Focused EMDR for PDs Among Outpatients [TEMPO] study). The trial protocol (Supplement 1) adhered to the Consolidated Standards of Reporting Trials (CONSORT) guidelines, was approved by the Medical Research Ethics Committee of the Erasmus Medical Center in Rotterdam, and was published previously.^22^ All participants provided written informed consent.

### Participants

A total of 159 participants were recruited from the outpatient clinics of 2 mental health care institutions in the Netherlands, the Parnassia Psychiatric Institute, the Hague, and GGZ Delfland, Delft. Eligible participants had to be 18 years or older and diagnosed with any PD using the Structured Clinical Interview for *DSM-5* Personality Disorders (SCID-5-PD).^23^ Patients diagnosed with other specified PD had to present with at least 10 PD symptoms. The exclusion criteria were inadequate competency in the Dutch language or an estimated IQ below 70. A power analysis^22^ determined that a total sample size of 132 participants was needed to detect a treatment effect on PD symptom severity using linear mixed models, assuming an effect size of Cohen *d* = 0.43, a correlation of 0.70 between repeated measures, α = .05, and power of 0.80. Accounting for 20% missing data, the final sample size was 159 participants.

### Measures

Assessments were conducted at baseline, post treatment, and at the 3-month follow-up. Assessors (including S.H.) underwent standardized external training sessions to ensure fidelity in administering the structured interviews. All outcome assessments were conducted blind to treatment allocation, and assessors were not involved in the treatment. The primary measure was the Assessment of *DSM-IV* PDs (ADP-IV), which provides a total severity score and allows for a categorical PD diagnosis.^24^ Secondary measures included the SCID-5-PD, a semistructured interview used for the evaluation of *DSM-5* PDs^23^; the Level of Personality Functioning Scale–Brief Form 2.0 (LPFS), which assesses personality functioning based on the alternative *DSM-5* model for PDs^25,26^; and the Difficulties in Emotion Regulation Scale (DERS), a self-report questionnaire developed to measure clinically relevant difficulties in emotion regulation.^27^ Hofman et al^22^ provide a description of the design and an overview of the measures.

### Procedure

Recruitment for this study was conducted between February 22, 2021, and April 9, 2024, with data collection completed on October 3, 2024. Participants were recruited via multiple routes: (1) posters in outpatient clinic waiting rooms, (2) direct referral by therapists, and (3) proactive outreach by researchers to patients who had agreed to be contacted for research purposes. Recruitment emphasized that individuals with any PD could participate, regardless of the presence of PTSD or traumatic memories. Interested individuals received detailed study information and were screened for eligibility by the research team prior to providing informed consent. Randomization was performed using a pregenerated random sequence (1:1 allocation without blocks) created in ResearchManager electronic data capture, an independent external platform used for secure data management. Allocation was concealed from blinded researchers, with only nonblinded researchers (including S.H. and L.H.) and the principal investigator (C.W.S.) having access to the allocation through a restricted interface; details of the process are available from Hofman et al.^22^ Participants in the waiting-list condition did not receive any trauma-focused or PD treatment and their medication regimens remained unchanged, although interim contact was possible if necessary. For all participants, the therapist who conducted the initial diagnostic assessment consulted with the participant after the 3-month follow-up to determine whether other treatments were necessary.

### Treatment

One-half of the participants received 10 EMDR sessions, delivered twice weekly during 5 weeks, with each session lasting 90 minutes. Treatment followed a structured case conceptualization.^7^ Sessions initially targeted traumatic memories meeting PTSD criterion A,^25^ prioritizing intrusive memories before nonintrusive memories. Therapy focused on memories associated with the patient’s most prominent PD symptoms. The standard EMDR protocol^28^ was used, while EMDR 2.0 incorporated a higher working memory load.^29^ Treatment-interfering anticipatory fear was addressed using the flashforward protocol.^30^ Participants who no longer met the PD diagnostic criteria based on the ADP-IV score during the treatment phase were defined as early completers.

### Supervision and Fidelity Monitoring

EMDR therapy was administered by certified therapists who had completed an accredited Dutch Basic EMDR training program. Prior to the study, all therapists attended an additional 1-day training led by an EMDR Europe Association (EMDREA)–accredited trainer. Each case conceptualization and EMDR session was reported by the therapists to an EMDREA-approved consultant using a custom-made form that included details about the target memories and their subjective unit of disturbance level before and after a therapy session, time spent on fully reprocessing the memories, and general session summaries. Monthly group supervision was provided by EMDREA-approved consultants, in which video recordings of EMDR sessions were presented. Additional supervision was provided on request. All sessions were videotaped, and for 20% of the participants, 1 session was randomly selected and rated by trained raters blinded to the treatment outcomes. Protocol adherence was high, with a score of 9.8 of 11 (89.5%); 100% of the sessions scored at least 8 points.

### Statistical Analysis

Data were analyzed using SPSS, version 27 (IBM Corporation) and R, version 4.3.3, with the nlme and lme4 packages (R Program for Statistical Computing). Primary and secondary outcomes were analyzed using linear mixed models (LMMs). Each model included fixed effects for group (EMDR vs control), time (baseline, post treatment, and follow-up), their interaction, and baseline severity, as well as a random intercept for participants and a random slope for time. Models were estimated using restricted maximum likelihood with an autoregressive covariance structure to account for temporal correlations between repeated measures. This approach incorporates all available data under the assumption of missing at random, allowing inclusion of all randomized participants in line with the intention-to-treat principle.

To aid interpretability, within-group and between-group Cohen *d* effect sizes were reported for descriptive purposes only. Within-group effect sizes reflect the magnitude of change over time within each condition, while between-group effect sizes post treatment and at follow-up indicate the size of group differences at those time points. Cohen *d* was calculated by dividing the mean difference (within or between groups) by the pooled SD. Effect sizes were categorized as small (0.20), medium (0.50), or large (0.80). A 2-tailed significance level of α = .05 was applied to all inferential tests.

Generalized LLMs (GLLMs) were used to test whether PD remission according to the ADP-IV was more common in the EMDR group than in the control group. In addition to the interaction term (group × time), all models included main effects of group (2 levels: EMDR vs control) and time (3 levels: baseline, post treatment, and follow-up) and a random effect controlling for within-participant variance. As SCID-5-PD diagnosis was required for inclusion, all participants (100%) had a PD at baseline, preventing convergence of GLMMs. Therefore, to compare PD remission based on the SCID-5-PD, χ^2^ tests were conducted with group (2 levels: EMDR vs control) and PD diagnosis (2 levels: yes vs no) post treatment and at 3-month follow-up, applying a Bonferroni correction for multiple testing. Odds ratios (ORs) were calculated to quantify the effect size.

Sensitivity analyses were conducted to examine the robustness of the findings, including completers only (SCID-5-PD, 122; ADP-IV, 113; LPFS, 107; and DERS, 107) and multiple imputation (MI) using the mice package in R, version 4.3.3.^31^ MI generated 35 datasets over 50 iterations, accounting for the multilevel data structure.^32^ Estimates and SEs were pooled using the Rubin rules.

## Results

As illustrated in the Figure, 159 participants (mean [SD] age, 35.4 [12.0] years; 130 female [81.8%] and 29 male [18.2%]) were randomly allocated to either EMDR therapy (n = 79) or a waiting-list control group (n = 80). Outcome data were available for 137 participants (86.2%) post treatment and 127 (79.9%) participants at follow-up. Table 1 presents the baseline demographic and clinical characteristics, which were well-balanced between the groups.

**Figure.**
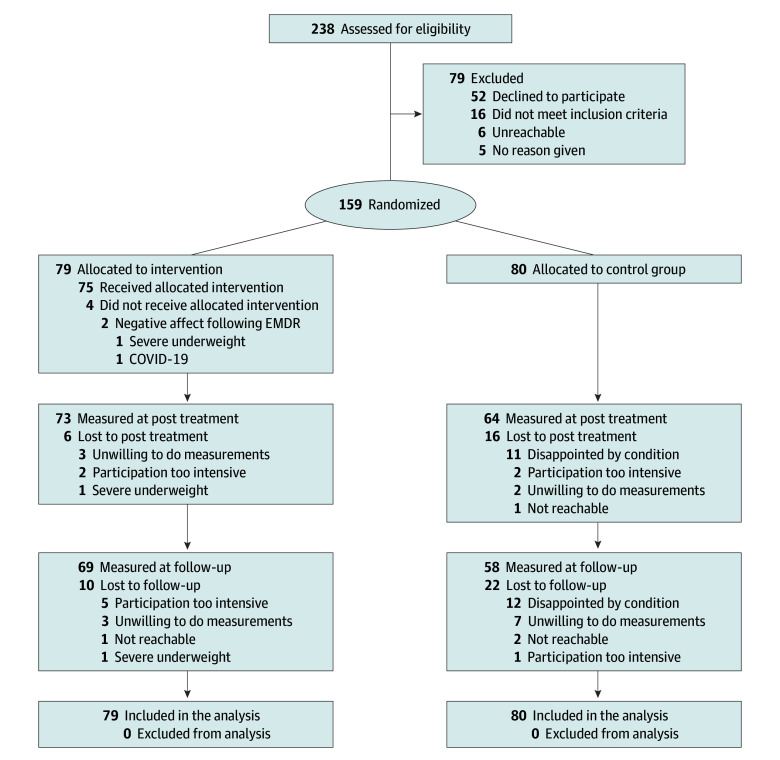
CONSORT Participant Flow Diagram EMDR indicates eye movement desensitization and reprocessing.

**Table 1.** Sample Characteristics

Characteristic	Participant group, No. (%)		
	Total (N = 159)	EMDR (n = 79)	Control (n = 80)
Age, mean (SD), y	35.4 (12.0)	34.3 (11.7)	36.5 (12.2)
Sex			
Female	130 (81.8)	64 (81.0)	66 (82.5)
Male	29 (18.2)	15 (19.0)	14 (17.5)
Race and ethnicity			
African	8 (5.0)	5 (6.3)	3 (3.8)
Asian	4 (2.5)	3 (3.8)	1 (1.3)
Eastern European	3 (1.9)	1 (1.3)	2 (2.5)
Latin American	18 (11.3)	7 (8.9)	11 (13.8)
Middle Eastern	3 (1.9)	2 (2.5)	1 (1.3)
Turkish	3 (1.9)	1 (1.3)	2 (2.5)
White and Western European	119 (74.8)	60 (75.9)	59 (73.8)
Missing	1 (0.6)	0	1 (1.3)
Working	82 (51.6)	40 (50.6)	42 (52.5)
Educational attainment			
None	2 (1.3)	1 (1.3)	1 (1.3)
Primary school	7 (4.4)	4 (5.1)	3 (3.8)
Secondary school	32 (20.1)	21 (26.6)	11 (13.8)
Secondary vocational	63 (39.6)	30 (38.0)	33 (41.3)
Higher professional	32 (20.1)	16 (20.3)	16 (20.0)
University	23 (14.5)	7 (8.9)	16 (20.0)
Single	84 (52.8)	47 (59.5)	37 (46.3)
Children	69 (43.4)	36 (45.6)	33 (41.3)
Previous treatment	143 (89.9)	70 (88.6)	73 (91.3)
Previous treatment duration, mean (SD), wk	102.6 (166.7)	102.3 (188.9)	102.9 (143.6)
Medications used	79 (49.7)	38 (48.1)	41 (51.3)
Antidepressants	62 (39.0)	27 (34.2)	35 (43.8)
Antipsychotics	17 (10.7)	8 (10.1)	9 (11.3)
Mood stabilizers	2 (1.3)	0	2 (2.5)
Benzodiazepines	27 (17.0)	13 (16.5)	14 (17.5)
Psychostimulants	14 (8.8)	10 (12.7)	4 (5.0)
Primary PD diagnosis^a^			
Avoidant PD	58 (36.5)	31 (39.2)	27 (33.8)
Dependent PD	2 (1.3)	1 (1.3)	1 (1.3)
Obsessive compulsive PD	17 (10.7)	7 (8.9)	10 (12.5)
Paranoid PD	3 (1.9)	1 (1.3)	2 (2.5)
Histrionic PD	1 (0.6)	1 (1.3)	0
Borderline PD	51 (32.1)	25 (31.6)	26 (32.5)
Antisocial PD	2 (1.3)	0	2 (2.5)
Other specified^b^	25 (15.7)	13 (16.5)	12 (15.0)
PTSD diagnosis^c^	62 (39.2)	32 (40.5)	30 (38.0)
PTSD criterion A trauma^c^	101 (63.9)	47 (59.5)	54 (68.4)

Abbreviations: EMDR, eye movement desensitization and reprocessing; PD, personality disorder.

^a^ Schizotypal, schizoid, and narcissistic PDs were not diagnosed in the sample.

^b^ Patients with other specified PDs presented with the following mean distribution of PD criteria: 2.3 borderline, 1.4 avoidant, 1.1 obsessive-compulsive, 1 paranoid, 0.6 dependent, 0.4 schizotypal, 0.2 schizoid, narcissistic, and histrionic, and 0.1 antisocial PD criteria. Additionally, 6 of 24 patients with other specified PD with an available CAPS-5 at baseline (25.0%) had PTSD.

^c^ Based on the Clinician-Administered PTSD scale for *DSM-5*, available for 79 participants in the EMDR group and 79 in the control group.

Of the 79 EMDR participants, 4 (5.1%) discontinued therapy. No adverse events were reported. A total of 25 participants (31.6%) fully processed all targeted memories before completing all EMDR sessions. Among them, 16 (20.3%) no longer met the diagnostic criteria for a PD according to the ADP-IV and were classified as early completers. In total, 25 participants (15.7%) deviated from the medication stabilization protocol. These deviations were evenly distributed between the EMDR (13 [16.5%]) and control (12 [15.0%]) groups.

### Intention-to-Treat Analyses

LLMs revealed significant group × time interaction effects across all primary and secondary outcomes (Table 2), indicating greater improvements in PD symptom severity, personality functioning, and emotion regulation in the EMDR group than in the control group. Specifically, EMDR therapy outperformed waiting list for ADP-IV post treatment (β, −37.93 [95% CI, −52.54 to −23.33]; *P* < .001; Cohen *d* = 0.31 [95% CI, −0.05 to 0.66]) and at follow-up (β, −45.73 [95% CI, −64.90 to −26.56]; *P* < .001; Cohen *d* = 0.46 [95% CI, 0.10-0.82]), SCID-5-PD post treatment (β, −3.65 [95% CI, −5.87 to −1.42]; *P* = .002; Cohen *d* = 0.48 [95% CI, 0.14-0.82]) and at follow-up (β, −3.70 [95% CI, −7.10 to −0.30]; *P* = .03; Cohen *d* = 0.61 [95% CI, 0.25-0.97]), LPFS post treatment (β, −3.13 [95% CI, −4.86 to −1.41]; *P* < .001; Cohen *d* = 0.31 [95% CI, −0.05 to 0.67]) and at follow-up (β, −3.62 [95% CI, −5.96 to −1.28]; *P* = .003; Cohen *d* = 0.43 [95% CI, 0.06-0.79]), and DERS post treatment (β, −9.03 [95% CI, −14.90 to −3.15]; *P* = .003; Cohen *d* = 0.35 [95% CI, −0.01 to 0.71]) and at follow-up (β, −11.73 [95% CI, −19.90 to −3.55]; *P* = .005; Cohen *d* = 0.62 [95% CI, 0.25-0.98]). Sensitivity analyses with completers only showed similar results. Site differences were not found to influence treatment outcomes, as indicated by model comparisons (χ^2^<0.01 for all; *P* = .99]) and interaction tests.

**Table 2.** Outcomes and Fixed-Effect Estimates of LMMs

Outcome	EMDR		Control		Results of LMMs			Between-group Cohen *d* (95% CI)^a^
	Mean (SD) score	Within-group Cohen *d* (95% CI)^a^	Mean (SD) score	Within-group Cohen *d* (95% CI)^a^	β (95% CI)	*t*	*P* value	
**Primary outcome**								
ADP-IV^b^								
Baseline	272.00 (62.32)	NA	263.17 (70.23)	NA	−0.81 (−9.34 to 7.73)	−0.18	.85	NA
Post treatment	222.36 (69.28)	0.76 (0.41 to 1.09)	244.28 (74.39)	0.26 (−0.08 to 0.60)	−37.93 (−52.54 to −23.33)	−5.09	<.001	0.31 (−0.05 to 0.66)
Follow-up	209.47 (72.86)	0.93 (0.58 to 1.12)	241.27 (63.31)	0.32 (−0.02 to 0.67)	−45.73 (−64.90 to −26.56)	−4.68	<.001	0.46 (0.10 to 0.82)
**Secondary outcomes**								
SCID-5-PD^c^								
Baseline	25.2 (9.23)	NA	27.39 (10.79)	NA	0.17 (−1.16 to 1.49)	0.25	.80	NA
Post treatment	20.88 (10)	0.45 (0.12 to 0.77)	25.72 (10.05)	0.16 (−0.17 to 0.49)	−3.65 (−5.87 to −1.42)	−3.21	.002	0.48 (0.14 to 0.82)
Follow-up	17.68 (10.59)	0.76 (0.42 to 1.09)	23.68 (8.7)	0.37 (0.03 to 0.71)	−3.7 (−7.10 to −0.30)	−2.13	.03	0.61 (0.25 to 0.97)
LPFS^d^								
Baseline	32.7 (5.82)	NA	32.92 (7)	NA	0.08 (−0.82 to 0.98)	0.18	.86	NA
Post treatment	27.79 (7.11)	0.76 (0.42 to 1.10)	29.93 (6.77)	0.43 (0.08 to 0.78)	−3.13 (−4.86 to −1.41)	−3.56	<.001	0.31 (−0.05 to 0.67)
Follow-up	26.25 (7.47)	0.98 (0.62 to 1.32)	29.34 (6.82)	0.52 (0.16 to 0.86)	−3.62 (−5.96 to −1.28)	−3.03	.003	0.43 (0.06 to 0.79)
DERS^e^								
Baseline	112.79 (21.39)	NA	114.64 (21.47)	NA	0.16 (−2.88 to 3.21)	0.11	.92	NA
Post treatment	101.17 (23.4)	0.52 (0.18 to 0.85)	109.13 (21.95)	0.25 (−0.10 to 0.60)	−9.03 (−14.90 to −3.15)	−3.01	.003	0.35 (−0.01 to 0.71)
Follow-up	95.81 (21.54)	0.79 (0.44 to 1.13)	108.88 (20.53)	0.27 (−0.08 to 0.62)	−11.73 (−19.90 to −3.55)	−2.81	.005	0.62 (0.25 to 0.98)

Abbreviations: ADP-IV, Assessment of *DSM-IV* PDs; DERS, Difficulties in Emotion Regulation Scale; EMDR, eye movement desensitization and reprocessing; LMM, linear mixed model; LPFS, Level of Personality Functioning Scale—Brief Form 2.0; NA, not applicable; SCID-5-PD, Structured Clinical Interview for *DSM-5* Personality Disorders.

^a^ Cohen *d* was calculated by dividing the mean difference by the pooled standard deviation, following the standard formula for comparison.

^b^ Scores range from 94 to 658, with higher scores indicating greater self-reported PD symptom severity.

^c^ Scores range from 0 to 158, with higher scores indicating greater clinician rated PD symptom severity.

^d^ Scores range from 12 to 48, with higher scores indicating lesser personality functioning.

^e^ Scores range from 36 to 180, with higher scores indicating greater difficulties in emotional regulation.

The initial MI included all available data (N = 159) and yielded consistent model estimates (β coefficients) compared with the primary analyses. However, reduced degrees of freedom in the MI led to lower power and higher *P* values, likely due to a subset of participants (n = 19) with baseline data only (primarily from the control group [n = 14]), EMDR dropouts (n = 4), and 1 early completer. This pattern suggests data may be missing not at random rather than missing completely at random. A second MI, excluding these 19 cases (n = 140), produced estimates, degrees of freedom, and *P* values closely in line with the primary analyses, supporting the robustness of the findings.

Between-group effect sizes were small post treatment (Cohen *d* = 0.31-0.48) and ranged from small to moderate at follow-up (Cohen *d* = 0.43-0.62) (Table 2). Within the EMDR group, improvements from baseline to post treatment were small to moderate (Cohen *d* = 0.45-0.76), increasing from baseline to follow-up to moderate to large (Cohen *d* = 0.76-0.98) (Table 2). Within the EMDR group, effect sizes varied from small to large across the diagnostic subgroups, but overall, indicated consistent benefits across different PDs (Table 3).

**Table 3.** Within-Group Cohen *d* for Subgroups Within EMDR Group^a^

Subgroup by timing of assessment	ADP-IV				SCID-5-PD				LPFS				DERS			
	Mean difference	Pooled SD	No. of participants	Cohen *d* (95% CI)	Mean difference	Pooled SD	No. of participants	Cohen *d* (95% CI)	Mean difference	Pooled SD	No. of participants	Cohen *d* (95% CI)	Mean difference	Pooled SD	No. of participants	Cohen *d* (95% CI)
Post treatment																
PTSD	52.71	67.74	29	0.78 (0.25 to 1.29)	6.47	9.74	29	0.66 (0.14 to 1.17)	4.89	6.54	29	0.75 (0.22 to 1.26)	12.21	24.2	29	0.50 (−0.01 to 1.01)
No PTSD	48.38	63.41	37	0.76 (0.31 to 1.20)	2.86	9.5	43	0.30 (−0.12 to 0.71)	4.91	6.45	37	0.76 (0.30 to 1.20)	11.58	20.25	37	0.57 (0.12 to 1.01)
Cluster B	41.34	72.57	45	0.57 (0.16 to 0.97)	2.9	11.63	47	0.25 (−0.15 to 0.64)	4.76	7	44	0.68 (0.27 to 1.08)	10.92	22.36	44	0.49 (0.08 to 0.89)
Cluster C	34.22	63.13	61	0.54 (0.19 to 0.88)	3.88	9.31	68	0.36 (0.03 to 0.69)	3	6.31	60	0.48 (0.13 to 0.82)	6.72	21.76	60	0.31 (−0.04 to 0.65)
Borderline PD	63.05	71.03	22	0.89 (0.27 to 1.47)	3.65	11.99	23	0.30 (−0.27 to 0.87)	5.69	7.21	22	0.79 (0.18 to 1.37)	17.34	20.67	22	0.84 (0.23 to 1.42)
Avoidant PD	46.48	52.76	27	0.88 (0.33 to 1.41)	4.17	9.37	29	0.44 (−0.07 to 0.95)	3.75	5.28	27	0.71 (0.17 to 1.23)	9.52	20.49	27	0.46 (−0.06 to 0.98)
Other specified PD	45.12	64.46	8	0.70 (−0.25 to 1.59)	4.26	5.96	10	0.63 (−0.24 to 1.45)	6.2	6.07	8	1.02 (0.01 to 1.94)	8.3	21.28	8	0.39 (−0.55 to 1.29)
Other PDs	37.06	77.98	9	0.48 (−0.46 to 1.37)	4.5	6.78	10	0.75 (−0.18 to 1.62)	5.52	7.66	9	0.72 (−0.24 to 1.61)	8.62	30.3	9	0.28 (−0.63 to 1.18)
Follow-up																
PTSD	70.93	70.87	25	1.00 (0.43 to 1.54)	8.86	9.74	29	0.85 (0.30 to 1.38)	7.49	7.04	24	1.06 (0.48 to 1.61)	23.13	23.58	24	0.98 (0.41 to 1.53)
No PTSD	56.43	64.66	39	0.87 (0.42 to 1.31)	6.58	9.51	42	0.69 (0.26 to 1.11)	5.83	6.32	39	0.92 (0.46 to 1.36)	12.67	19.69	38	0.64 (0.19 to 1.08)
Cluster B	58.72	70.58	44	0.83 (0.41 to 1.24)	6.99	11.15	45	0.63 (0.22 to 1.03)	5.9	6.99	43	0.84 (0.42 to 1.25)	14.01	21.3	43	0.66 (0.24 to 1.06)
Cluster C	37.01	63.69	57	0.58 (0.23 to 0.93)	5.75	9.23	61	0.62 (0.28 0.96)	4.48	6.41	57	0.70 (0.34 to 1.05)	12.36	20.71	57	0.60 (0.24 to 0.95)
Borderline PD	78.5	71.61	21	1.10 (0.46 to 1.70)	8.32	11.45	21	0.73 (0.12 to 1.31)	6.93	7.59	20	0.91 (0.28 to 1.51)	20	20.81	20	0.96 (0.32 to 1.56)
Avoidant PD	67.13	56.82	26	1.18 (0.60 to 1.73)	7.34	9.76	29	0.75 (0.22 to 1.27)	6.53	5.81	26	1.12 (0.55 to 1.67)	18.29	19.32	26	0.95 (0.38 to 1.48)
Other specified PD	40.78	71.68	9	0.57 (−0.33 to 1.43)	5.96	9.63	10	0.62 (−0.25 to 1.44)	4.78	6.59	9	0.73 (−0.21 to 1.60)	3.11	24.04	9	0.13 (−0.76 1.01)
Other PDs	35.75	77.57	8	0.46 (−0.46 to 1.37)	6.17	6.25	8	0.99 (−0.04 to 1.92)	6.93	6.79	8	1.02 (−0.01 to 1.95)	21.28	25.96	8	0.82 (−0.18 to 1.74)

Abbreviations: ADP-IV, Assessment of *DSM-IV* PDs; DERS, Difficulties in Emotion Regulation Scale; LPFS, Level of Personality Functioning Scale—Brief Form 2.0; PD, personality disorder; PTSD, posttraumatic stress disorder; SCID-5-PD, Structured Clinical Interview for *DSM-5* Personality Disorders.

^a^ Within-group effect sizes reflect the magnitude of change from baseline to either post treatment or follow-up. Cluster A Cohen *d*s could not be calculated due to lack of data.

A GLLM revealed a significant group × time interaction, with a greater reduction in ADP-IV diagnoses in the EMDR group compared with the control group from baseline to post treatment (38.3% vs 6.8%; β = −2.27 [95% CI, −3.81 to −0.73]; *P* = .003; OR, 0.10 [95% CI, 0.02-0.47]) and from baseline to 3-month follow-up (45.4% vs 5.9%; β = −2.90 [95% CI, −4.51 to −1.29]; *P* < .001; OR, 0.06 [95% CI, 0.01-0.27]). SCID-5-PD diagnoses also declined more in the EMDR group than in the control group, both post treatment (24 [33.3%] vs 5 [7.8%]; χ^2^_1_ = 11.68 [n = 136]; *P* < .001; OR, 0.17 [95% CI, 0.06-0.48]) and at follow-up (30 [44.1%] vs 9 [15.8%]; χ^2^_1_, = 10.31 [n = 125]; *P* = .001; OR, 0.24 [95% CI, 0.10-0.56]) (Table 4). Sensitivity analyses of completer only cases showed similar results.

**Table 4.** PD Diagnoses Per Subgroup Per Time Point

Measure by timing of assessment	Control group (n = 80)			EMDR group (n = 79)			Test statistic^a^		
	No. with PD	No. with no PD	% With PD	No. with PD	No. with no PD	% With PD	β (95% CI) or χ^2^	*P* value	OR (95% CI)
**All subgroups**									
ADP-IV									
Baseline	57	20	74.0	67	11	85.9	β = 1.13 (−0.17 to 2.43)	.08	3.09 (0.86 - 11.06)
Post treatment	40	18	69.0	35	31	53.0	β = −2.27 (−3.81 to −0.73)	.003	0.10 (0.02-0.47)
Follow-up	39	17	69.6	30	34	46.9	β = −2.90 (−4.51 to −1.29)	<.001	0.06 (0.01-0.27)
SCID-5-PD									
Baseline	80	0	100	79	0	100	NA	NA	NA
Post treatment	59	5	92.2	48	24	66.7	χ^2^_1_, = 11.68	.001	0.17 (0.06- 0.48)
Follow-up	48	9	84.2	38	30	55.9	χ^2^_1_, = 10.31	.001	0.24 (0.10-0.56)
**PTSD subgroup**									
ADP-IV									
Baseline	22	7	75.9	28	4	87.5	β = 1.14 (−0.84 to 3.12)	.25	3.12 (0.44-21.95)
Post treatment	12	6	66.7	16	13	55.2	β = −1.88 (−4.28 to 0.52)	.13	0.15 (0.01-1.68)
Follow-up	12	7	63.2	13	12	52.0	β = −2.06 (−4.52 to 0.40)	.10	0.13 (0.01-1.47)
SCID-5-PD									
Baseline	30	0	100	32	0	100	NA	NA	NA
Post treatment	19	0	100	20	9	69.0	NA^b^	.007	0 (0-0.64)
Follow-up	17	3	85.0	15	11	57.7	NA^b^	.09	0.24 (0.06-1.03)
**No PTSD subgroup**									
ADP-IV									
Baseline	35	13	72.9	39	7	84.8	1.10 (−0.59 to 2.80)	.20	3.01 (0.55-16.32)
Post treatment	28	12	70.0	19	18	51.4	−2.51 (−4.51 to −0.51)	.01	0.08 (0.01-0.59)
Follow-up	17	10	63.0	17	22	43.6	−3.45 (−5.57 to −1.33)	.001	0.03 (<0.01-0.27)
SCID-5-PD									
Baseline	49	0	100	47	0	10v0	NA	NA	NA
Post treatment	40	5	88.9	28	15	65.1%	χ^2^_1_, = 5.79	.02	0.23 (0.08-0.72)
Follow-up	31	6	83.8	23	19	54.8	χ^2^_1_, = 6.38	.01	0.23 (0.08-0.68)
**Borderline PD subgroup**									
ADP-IV									
Baseline	23	5	82.1	21	4	84.0	β = −0.76 (−2.27 to 0.75)	.55	0.47 (0.04-5.60)
Post treatment	20	1	95.2	10	12	45.5	β = −4.83 (−8.97 to −0.69)	.02	0.01 (<0.01-0.49)
Follow-up	16	4	80.0	8	13	38.1	β = −2.93 (−5.06 to −0.80)	.06	0.05 (<0.01-1.18)
SCID-5-PD									
Baseline	26	0	100	25	0	100	NA	NA	NA
Post treatment	21	1	95.5	16	7	69.6	NA^b^	.047	0.11 (0.01-0.98)
Follow-up	16	5	76.2	9	12	42.9	NA^b^	.06	0.23 (0.06-0.88)
**Avoidant PD subgroup**									
ADP-IV									
Baseline	21	3	87.5	30	1	96.8	β = 1.63 (−1.14 to 4.39)	.24	5.13 (0.33-78.73)
Post treatment	14	5	73.7	20	7	74.1	β = −1.73 (−4.80 to 1.34)	.27	0.18 (0.01-3.77)
Follow-up	15	3	83.3	14	12	53.8	β = −3.80 (−7.14 to −0.46)	.03	0.02 (<0.01-0.61)
SCID-5-PD									
Baseline	27	0	100	31	0	100	NA	NA	NA
Post treatment	20	1	95.2	21	8	72.4	NA^b^	.06	0.13 (0.02-1.15)
Follow-up	17	2	89.5	19	10	65.5	NA^b^	.09	0.22 (<0.01-0.61)
**Other specified PD subgroup**									
ADP-IV									
Baseline	5	7	41.7	8	4	66.7	β = 1.92 (−0.10 to 3.94)	.21	6.83 (0.34-136.33)
Post treatment	3	6	33.3	2	6	25.0	β = −2.20 (−6.02 to 1.61)	.25	0.11 (<0.01-4.68)
Follow-up	4	4	50.0	4	5	44.4	β = −2.57 (−6.47 to 1.33)	.20	0.08 (<0.01-3.79)
SCID-5-PD									
Baseline	12	0	100	13	0	100	NA	NA	NA
Post treatment	9	1	90.0	5	5	50.0	NA^b^	.14	0.11 (0.01-1.24)
Follow-up	6	1	85.7	6	4	60.0	NA^b^	.34	0.25 (0.02-2.94)
**Other PD subgroup**									
ADP-IV									
Baseline	8	7	53.3	8	2	80.0	β = 2.59 (−1.23 to 6.41)	.18	13.29 (0.30-593.77)
Post treatment	3	6	33.3	3	6	33.3	β = −2.13 (−6.46 to 2.21)	.33	0.12 (<0.01-8.97)
Follow-up	4	6	40.0	4	4	50.0	β = −1.71 (−5.81 to 2.39)	.42	0.18 (<0.01-11.11)
SCID-5-PD									
Baseline	15	0	100	10	0	100	NA	NA	NA
Post treatment	9	2	81.8	6	4	60.0	NA^b^	.36	0.33 (0.05-2.43)
Follow-up	9	1	90.0	4	4	50.0	NA^b^	.12	0.11 (0.01-1.34)

Abbreviations: Abbreviations: ADP-IV, Assessment of *DSM-IV* PDs; NA, not applicable; PD, personality disorder; PTSD, posttraumatic stress disorder; SCID-5-PD, Structured Clinical Interview for *DSM-5* Personality Disorders.

^a^ Calculated as results of generalized linear mixed models for ADP-IV diagnoses and χ^2^ analyses of SCID-5-PD diagnoses.

^b^ Considering the small sample size, Fisher exact test was performed, which does not rely on a test statistic.

## Discussion

This randomized clinical trial evaluated the effectiveness of standalone EMDR therapy in the treatment of PDs compared with a waiting-list control condition, regardless of comorbid PTSD. The results demonstrated significant reductions in PD symptom severity post treatment and at 3-month follow-up, with diagnostic remission achieved in 44.1% of participants, and improvements in personality functioning and emotion regulation. These effects were consistent across PD subtypes and among patients with and without baseline PTSD diagnosis. Treatment drop-out was low (4 [5.1%]), and no adverse events were reported.

These findings contribute to a growing body of evidence supporting trauma-focused interventions in the treatment of PDs,^9,11^ including standalone approaches.^12,13,14,15^ The results align with a prior study demonstrating that trauma-focused therapy can be effective in PD populations even in the absence of PTSD.^8^ The present study extends the literature by systematically examining PD symptom severity, diagnostic remission, and emotion dysregulation, providing robust evidence for the safe and effective use of EMDR therapy as a standalone intervention for PDs irrespective of comorbid PTSD. While previous studies^7,9,10^ have predominantly focused on the treatment of borderline PD, often using long-term psychotherapeutic interventions, the present study suggests that targeting memories of traumatic and adverse events through EMDR may produce clinically meaningful improvements across a range of PDs. The results align with theoretical models that conceptualize maladaptive personality traits as stemming from unresolved traumatic experiences and reinforce the emerging view that trauma-focused therapies can be beneficial beyond populations with traditional PTSD.^7^

### Recommendations

These findings suggest that EMDR therapy may serve as a useful starting point in the treatment of PDs, particularly considering the substantial reductions in PD symptom severity and diagnostic status observed after a brief intervention. Given the continued improvement found at follow-up, it may be appropriate to re-evaluate the need for additional treatment 3 months after therapy. While some patients may benefit sufficiently from EMDR therapy alone, others may require further interventions. Future research should assess long-term effects of EMDR and compare these with active controls, such as standard PD treatments and alternative trauma-focused treatments. This could help clarify the specific contribution of EMDR therapy and mitigate potential expectancy effects. To maximize therapeutic outcomes, the optimal delivery of EMDR therapy should be investigated by examining variables such as session number, frequency, and timing.

### Strengths and Limitations

This study has several strengths, including high retention rates, use of both dimensional and categorical assessments of PDs, participation of individuals with and without PTSD, and a broad range of PDs, including less commonly studied types, such as avoidant and other specified PDs, thereby enhancing the ecological validity of the findings. The effect sizes provided quantitative benchmarks for comparison with previous research.

Certain limitations should also be noted. First, a waiting-list control condition limits not only the ability to attribute treatment effects specifically to EMDR therapy, but also the capacity to estimate its true effect size. Recent meta-analytic evidence from depression research^33^ indicates that trials using waiting-list control groups tend to yield inflated effect sizes compared with those using care-as-usual controls, largely due to the lower pretreatment-posttreatment change observed in the waiting-list conditions themselves. In addition, the use of a waiting-list group may exert expectancy effects in that participants know they will receive the intervention eventually, which could mitigate observed differences between groups. Nevertheless, we concur that future trials in this field should aim to include active or usual-care control conditions to increase the internal and external validity of findings. Second, double-blinding (eg, sham-EMDR) was not feasible, potentially introducing expectancy effects. Third, the control group showed significant improvements over time as well, possibly reflecting the impact of study participation itself. Fourth, although the diagnostic instruments used in this study (ie, ADP-IV and SCID-5-PD) are designed to assess enduring personality pathology, the 3-month follow-up remains a relatively short timeframe to evaluate sustained change. Fifth, although the sample included a broad range of PD types, most participants had avoidant, borderline, obsessive-compulsive, or other specified PDs, limiting generalizability to less prevalent PD types. Last, while the benefits persisted for at least 3 months, the long-term effects remain unknown.

## Conclusions

This randomized clinical trial supports the potential of trauma-focused approaches, such as EMDR therapy, in the treatment of personality pathology. Further research is warranted to confirm and extend these results, including head-to-head comparisons with established psychotherapeutic interventions for PDs.

## Notes

Supplement 1.
Trial Protocol

Supplement 2.
Data Sharing Statement
