# Supplementary material for: Eye Movement Desensitization and Reprocessing Therapy in Persons With Personality Disorders: A Randomized Clinical Trial
Source: JAMA Netw Open. 2025 Sep 25;8(9):e2533421. doi: 10.1001/jamanetworkopen.2025.33421 (PMC12464786; doi:10.1001/jamanetworkopen.2025.33421)
Supplement: Supplement 1. — Trial Protocol [file jamanetwopen-e2533421-s001.pdf]

Trauma-focused EMDR for Personality disorders among Outpatients
(Versie 6, 19 Januari 2021)

- May 2015: adaptation section 11.5: text in accordance to old and new Measure regarding Compulsory Insurance for Clinical Research in Humans
- Sept 2015: adaptation section 9.1, 9.2 and 12.5: text in accordance to WMO amendment on reporting SAE and temporary halt (section 10 of WMO)
- Oct 2015: adaptation section 4.4 – comment [CCMO15], 8.2 and 10.1 with respect to methodology/statistics
- Sept 2018: adaptation section 12.1 and comment [CCMO46] due to applicability GDPR as of May, 2018

PROTOCOL TITLE

Trauma-focused EMDR for Personality disorders among Outpatients

**Protocol ID**	**NL73628.078.20**
**Short title**	**Trauma-focused EMDR for PD**
**EudraCT number**	***Not applicable***
**Version**	**6**
**Date**	**19-01-2021**
**Coordinating investigator/project leader**	***MSc, S. Hofman, Department of Personality Disorders, Lijnbaan 4, 2512VA, The Hague, the Netherlands, 0883573107, s.hofman@parnassiagroep.nl***
**Principal investigator(s) (in Dutch: hoofdonderzoeker/ uitvoerder)**	***Prof. dr. C. W. Slotema, Department of Personality Disorders, Lijnbaan 4, 2512VA, The Hague, the Netherlands, 0883573107, c.slotema@psyq.nl***<br>***MSc, L. C. S. Hafkemeijer, GGZ Delfland, Delft, L.Hafkemeijer@ggz-delfland.nl***
**Sponsor (in Dutch: verrichter/opdrachtgever)**	***Prof. dr. C.W. Slotema, senior researcher Department of Personality Disorders***
**Subsidising party**	
**Independent expert**	***Drs. W. Pardoen, MD***<br>***Lijnbaan 4, 2512VA4, The Hague, the Netherlands,***<br>***w.pardoen@psyq.nl***
**Laboratory sites**	**-**

**Pharmacy**	**-**

PROTOCOL SIGNATURE SHEET

**Name**	**Signature**	**Date**
**Head of Department:**<br>**M. Wesseling**<br>**Directeur zorg I-psy, PsyQ**<br>**Brijder**	m wesseling	15-12-'20
**Coordinating Investigators**<br>**C.W. Slotema**<br><br>**L.C.S. Hafkemeijer**		**10-12-2020**<br><br>**10-12-2020**

TABLE OF CONTENTS

1. INTRODUCTION AND RATIONALE 9
2. OBJECTIVES 13
3. STUDY DESIGN 14
4. STUDY POPULATION 15
 4.1 Population 15
 4.2 Inclusion criteria 15
 4.3 Exclusion criteria 15
 4.4 Sample size calculation 15
5. TREATMENT OF SUBJECTS 16
 5.1 Investigational product/treatment 16
 5.2 Use of co-intervention 16
 5.3 Escape medication 16
6. INVESTIGATIONAL PRODUCT 17
 6.1 Name and description of investigational product(s) 17
 6.2 Summary of findings from non-clinical studies 17
 6.3 Summary of findings from clinical studies 17
 6.4 Summary of known and potential risks and benefits 17
 6.5 Description and justification of route of administration and dosage 17
 6.6 Dosages, dosage modifications and method of administration 17
 6.7 Preparation and labelling of Investigational Medicinal Product 17
 6.8 Drug accountability 17
7. NON-INVESTIGATIONAL PRODUCT 18
8. METHODS 19
 8.1 Study parameters/endpoints 19
  8.1.1 Main study parameter/endpoint 19
  8.1.2 Secondary study parameters/endpoints (if applicable) 19
  8.1.3 Other study parameters (if applicable) 19
 8.2 Randomisation, blinding and treatment allocation 20
 8.3 Study procedures 20
 8.4 Withdrawal of individual subjects 21
 8.4.1 Specific criteria for withdrawal (if applicable) 21
 8.5 Replacement of individual subjects after withdrawal 21
 8.6 Follow-up of subjects withdrawn from treatment 21
 8.7 Premature termination of the study 21
9. SAFETY REPORTING 22
 9.1 Temporary halt for reasons of subject safety 22
 9.2 AEs, SAEs and SUSARs 22
  9.2.1 Adverse events (AEs) 22
  9.2.2 Serious adverse events (SAEs) 22
  9.2.3 Suspected unexpected serious adverse reactions (SUSARs) 23
 9.3 Annual safety report 24

9.4 Follow-up of adverse events 24
9.5 [Data Safety Monitoring Board (DSMB) / Safety Committee] 25
10. STATISTICAL ANALYSIS 26
10.1 Primary study parameter(s) 26
10.2 Secondary study parameter(s) 26
10.3 Other study parameters 27
10.4 Interim analysis 27
11. ETHICAL CONSIDERATIONS 28
11.1 Regulation statement 28
11.2 Recruitment and consent 28
11.3 Objection by minors or incapacitated subjects 28
11.4 Benefits and risks assessment, group relatedness 28
11.5 Compensation for injury 29
11.6 Incentives (if applicable) 29
12. ADMINISTRATIVE ASPECTS, MONITORING AND PUBLICATION 30
12.1 Handling and storage of data and documents 30
12.2 Monitoring and Quality Assurance 30
12.3 Amendments 30
12.4 Annual progress report 31
12.5 Temporary halt and (prematurely) end of study report 31
12.6 Public disclosure and publication policy 31
13. STRUCTURED RISK ANALYSIS 32
13.1 Potential issues of concern 32
13.2 Synthesis 33
14. REFERENCES 34

LIST OF ABBREVIATIONS AND RELEVANT DEFINITIONS

ABR	General Assessment and Registration form (ABR form), the application form that is required for submission to the accredited Ethics Committee; in Dutch: Algemeen Beoordelings- en Registratieformulier (ABR-formulier)
AE	Adverse Event
AR	Adverse Reaction
CA	Competent Authority
CCMO	Central Committee on Research Involving Human Subjects; in Dutch: Centrale Commissie Mensgebonden Onderzoek
CV	Curriculum Vitae
DSMB	Data Safety Monitoring Board
	European Union
EMDR	Eye Movement Desentization and Reprocessing
EU	
EudraCT	European drug regulatory affairs Clinical Trials
GCP	Good Clinical Practice
GDPR	General Data Protection Regulation; in Dutch: Algemene Verordening Gegevensbescherming (AVG)
IB	Investigator’s Brochure
IC	Informed Consent
IMP	Investigational Medicinal Product
IMPD	Investigational Medicinal Product Dossier
METC	Medical research ethics committee (MREC); in Dutch: medisch-ethische toetsingscommissie (METC)
PD	Personality Disorder
PTSD	Posttraumatic Stress Disorder
RCT	Randomized controlled trial
(S)AE	(Serious) Adverse Event
SPC	Summary of Product Characteristics; in Dutch: officiële productinformatie IB1-tekst
Sponsor	The sponsor is the party that commissions the organisation or performance of the research, for example a pharmaceutical

	**company, academic hospital, scientific organisation or investigator. A party that provides funding for a study but does not commission it is not regarded as the sponsor, but referred to as a subsidising party.**
**UAVG**	**Dutch Act on Implementation of the General Data Protection Regulation; in Dutch: Uitvoeringswet AVG**
**WMO**	**Medical Research Involving Human Subjects Act; in Dutch: Wet Medisch-wetenschappelijk Onderzoek met Mensen**

SUMMARY

Rationale: There is evidence suggesting that EMDR therapy is not only effective in the treatment of patients with personality disorders (PD) with and without PTSD, but also efficient, and thus cost-effective. Interventions for PD have a long duration and approximately 50% does not benefit sufficiently. The next step is to conduct a RCT for patients with all types of PD to explore the efficacy and cost-effectiveness of EMDR compared to waiting list to confirm these preliminary results.

Objective: The aim of this study is to evaluate the effectiveness of EMDR in reducing PD symptoms.

Study design: A multicenter, single-blind randomized controlled trial (RCT) with two arms: EMDR and no treatment (waiting list).

Study population: Patients with PD of eighteen years or older

Intervention (if applicable): The Dutch version of the standard EMDR protocol (de Jongh & ten Broeke, 2019) will be used. The protocol will focus on the most distressing target memory. A total of 10 biweekly sessions of EMDR will be applied with a duration of 90 minutes per session.

Main study parameters/endpoints: Mixed models, cost-utility and –effectiveness analyses will be conducted. The following outcome measures will be used: The Assessment van DSM Persoonlijkheidsstoornissen-IV and Clinician-Administered PTSD Scale for DSM-5 (primary outcomes) and the Structured Clinical Interview for DSM 5 Personality Disorders, Jeugd Trauma Vragenlijst, Life Events Checklist for the DSM-5, Level of Personality Functioning Scale - Brief Form 2.0, Clinician-Administered PTSD Scale for DSM-5, PTSD Check List, , Difficulties in Emotion Regulation Scale, Brief State Paranoia Checklist, Outcome Questionnaire-45, Euro-Quality of Life 5 dimension 5 level version, Treatment inventory Cost In Psychiatric Patients, Mental Health Quality of Life questionnaire.

Data will be collected at baseline, 5 week (post EMDR-treatment) and 3, 6 and 12 months after onset of the study.

Nature and extent of the burden and risks associated with participation, benefit and group relatedness: It is expected that patients who receive EMDR, will experience a reduction in PD- and PTSD-symptoms and an improvement in quality of life and interpersonal functioning. In addition, EMDR is safe and not associated with adverse events. The study requires participants in the control group to not receive treatment for three months. In the two or three days after an EMDR-session symptoms of emotion dysregulation or psychotrauma may increase. This effect is temporarily and not associated with any increase in selfinjurious – or suicidal behavior.

1. INTRODUCTION AND RATIONALE

Statement of the problem

Prevalence rates for childhood trauma among patients with personality disorders (PD) are very high. Verbal -, physical - and sexual abuse during childhood have been reported in, respectively, 72%, 46% and 26% of patients with borderline personality disorder (BPD, Zanarini, Gunderson, Marino, Schwartz, & Frankenburg, 1989). A total of ninety-seven percent of BPD patients were found to have experienced at least one type of childhood trauma, i.e., emotional -, physical – or sexual abuse, or emotional – and physical neglect at our department. Among different types of PD high prevalence rates were reported for abuse (73%) and neglect (82%, Battle et al., 2004). Estimates of the prevalence of PTSD in patients with BPD range 34% to 56% (Slotema, Blom, Niemantsverdriet, Deen & Sommer, 2018; Zanarini et al., 1998). For patients with other PD than BPD prevalence rates for PTSD vary between 20 and 47% (Goldstein et al., 2017; Zanarini et al., 1998; Yen et al., 2002). Psychotherapy is the treatment of first choice for patients with PD. However, training of therapists and the long duration of treatment are accompanied with high costs. Furthermore, symptoms are found to remain unchanged or exacerbate during psychotherapy for PD in 34 up to 57% (Giesen-Bloo et al., 2006; Svartberg, Stiles, & Seltzer, 2004; own data from clinical practice). Given these concerns, the high prevalence of trauma and associated consequences, the question arises whether treatment of patients with PD can be improved if interventions will be used that focus on trauma memories. There is mounting evidence suggesting that EMDR therapy is not only effective in the treatment of PD with and without PTSD but also efficient, and thus cost-effective. The need to improve the treatment of PD with interventions that focus on trauma is clear. The next step is to conduct a sufficiently powered RCT for patients with all types of PD to explore the efficacy and cost-effectiveness of EMDR compared to waiting list to confirm these preliminary results and to determine the effects in terms of loss of diagnoses and cost-saving one year following treatment.

Background

Personality disorders (PD) occur in 4-13% of the North American and European population (Tyrer, Reed, & Crawford, 2015), and 40-50% of patients in mental health care settings meet the diagnostic criteria for PD (Newton-Howes et al., 2010). Patients with PD suffer from low self-esteem and have severe impairments in the regulation of emotions and interpersonal functioning. Their quality of life is low and societal costs are high. Most studies in this area included patients with borderline personality disorder (BPD), in which PTSD is present in 34 up to 56% (Slotema et al., 2018; Zanarini et al., 1998). Patients with BPD and PTSD have a lower quality of life, more comorbid disorders and a higher risk for suicide attempts than patients with only one of those diagnoses. Although up to 66% of patients with BPD do not

fulfill the criteria for PTSD, 97% of them were found to have experienced at least one type of childhood trauma, including abuse and neglect (own data). Patients who have suffered from childhood trauma are in risk to have dysregulation of their emotions, hallucinations, suspiciousness, difficulties in attachment and to be revictimized during their lives (Niemantsverdriet et al., 2017; Zanarini, 2000; Voestermans et al., 2020; Seo & Choi, 2018). Although less extensively investigated, Battle and colleagues (2004) found that rates of childhood maltreatment among individuals with different types of PD are high, with 73% of their sample reporting abuse and 82% reporting neglect. Prevalence rates for PTSD among other PD than BPD range 20 to 47% (Goldstein et al., 2017; Zanarini et al., 1998; Yen et al., 2002).

Multidisciplinairy treatment guidelines state that cognitive behavioral therapy (CBT) and eye movement desensitization and reprocessing (EMDR) therapy are the treatments of choice for PTSD (de Jongh, Amann, Hofmann, Farrell, & Lee, 2019). A meta-analysis of four RCTs with psychotherapy (other than EMDR therapy) for PTSD in BPD revealed effect sizes ranging .54-.82 and the interventions were safe (Slotema, Arends, & Franken, submitted). Results for the influence of psychotherapy for PTSD on symptoms of PD are inconclusive (see for example Markowitz et al., 2015 and Kredlow et al., 2017). The same holds true for EMDR therapy in that the effects of EMDR on interpersonal functioning has barely been explored with one RCT revealing negative results for social functioning among patients with chronic psychotic disorders and comorbid PTSD (de Bont et al., 2016).

Although the literature suggests that EMDR therapy is more efficient, cost-effective and better tolerated than other interventions (including CBT; McQuire, Lee, & Dummond, 2014; de Jongh et al., 2019; Mavranezouli et al., 2020), EMDR has not been studied thoroughly in populations with PD. In an open study EMDR appeared to be beneficial and safe in patients with PD for PTSD (Slotema, van den Berg, Driessen, Wilhelmus, & Franken, 2019). No differences in severity of PTSD or efficacy of EMDR could be found between patients with BPD and other PD. De Jongh and colleagues (2020) established a significant reduction in PTSD symptoms with a combination of EMDR, prolonged exposure therapy, physical activity, and psychoeducation without any form of stabilization within only 8 treatment days. Of interest is that BPD symptoms decreased and one third of the patients lost their positive screen for BPD posttreatment. Furthermore, results of an RCT comparing five sessions of EMDR therapy to waiting list in patients diagnosed with PD without PTSD revealed medium to large effect sizes for psychological symptoms, psychological functioning, and personality functioning without any adverse events (Hafkemeijer, de Jongh, van der Palen, & Starrenburg, submitted). These findings are particularly important because the length of treatment was brief in that patients received only a total of 7.5 hours of EMDR therapy, whereas the gains were maintained at the end of the study, i.e., at 3-month follow-up. This is

remarkably shorter than in previous studies on the treatment of BPD, including Harned, Korslund, & Linehan (2014) whose patients received one full year of dialectical behavior therapy (DBT), existing of weekly sessions of individual therapy and group skills training later combined with the DBT prolonged exposure protocol, and has a much lower intensity of treatment than a 12-week residential of DBT, music – and art therapy, PTSD-specific psychoeducation and trauma-focused CBT (Bohus et al., 2013).

Significance of the proposed study

This study fits within the first priority – Advancing Evidence Based Practice – of the EMDR Foundation. This study will increase the availability of quality EMDR research in the field of PD. Although the efficacy of EMDR was explored in a number of studies, more evidence is needed to implement EMDR in the treatment of patients with PD. Furthermore, this study might result in the addition of EMDR to the guidelines for treatment of (some types of) PD. Therefore, the study has the potential to arrange a breakthrough in the treatment of patients with PD worldwide.

Value for clinical practice

PD are prevalent and ensuing distress is high for the patients as well as their surroundings. Evidence suggests that patients with PD might be even more stigmatized than other psychiatric diagnoses, with both fear and frustration among the common public reactions to personality disorders (Sheehan, Nieweglowski & Corrigan, 2016). The belief that people with personality disorders should be able to exhibit control over their behavior results in symptoms being viewed as manipulations or rejections of help. However, almost all of these patients are traumatized, suffer from bad memories, severe mood swings, suspiciousness and difficulties to attach to others.

Treatment of PD has a long duration and is expensive. The number of therapists is insufficient. As a result, many patients are waiting for treatment. Furthermore, approximately half of these patients do not benefit from these interventions with the risk that patients drop out from treatment.

Another concern is that symptoms of trauma and PTSD can be easily missed or delayed at a department for PD. Caregivers might be used to focus on the symptoms of PD and patients might be ashamed to talk about their traumatic experiences. In clinical practice interventions for trauma are often just considered if treatment for PD stagnates. By conducting this study, we also aim to improve the attention for and recognition of symptoms of trauma and PTSD in patients.

EMDR therapy can be applied within a short period, i.e., a couple of weeks. it has proven to be safe and is the most cost-effective trauma-focused treatment (Mavranezouli et al., 2020).

In addition, the literature suggests that EMDR not only decreases the severity of symptoms of trauma, but also symptoms of PD. If these findings can be confirmed in this RCT, EMDR might result in a decline of the duration of treatment of PD, the number of dropouts, waiting lists and societal costs.

Scientific value

Members of the project group are internationally considered as experts in the field of EMDR, PD, and cost-effectiveness studies. Although EMDR is the treatment of choice for PTSD, the literature regarding EMDR for PTSD among patients with PD is limited (Slotema et al., 2019 de Jongh et al., 2020). The same holds true for the efficacy of EMDR on the severity of symptoms of PD, presence of PD (de Jongh et al., 2020; Hafkemeijer et al., submitted) as well as interpersonal functioning. Cost-effectiveness studies are lacking. A RCT exploring differences between EMDR therapy and waiting list for the severity and presence of PD, trauma and cost-effectiveness is therefore urgently needed.

In addition, all RCTs investigating psychotherapy for PTSD are conducted with patients with BPD. Among open label studies only a minority of studies included patients with other PD. In clinical practice, patients with BPD comprise approximately 30% of the caseloads. It is of importance to pay more attention to other PD. By including patients with all types of PD, this study adds to the knowledge of treatment of other PD as well.

Finally, researchers in the field of other psychiatric disorders than PD might profit from this study as trauma is prevalent among other psychiatric disorders as well.

In order to inform other researchers about the procedure and results of this study, this study will be registered at www.clinicaltrial.gov.

2. OBJECTIVES

Primary Objectives:

1) How effective is EMDR therapy compared to waiting list on symptoms of PD?
2) How cost-effective is EMDR therapy versus a wait-list control condition?

Secondary Objectives:

3) What is the decline in the proportion of patients fulfilling a diagnosis of PD after EMDR?
4) How effective is EMDR therapy compared to waiting list on the severity of symptoms of PTSD?
5) How effective is EMDR therapy on mental health outcome, severity of difficulties in emotion regulation and interpersonal functioning?
6) What is the effectiveness of EMDR on quality of life?

3. STUDY DESIGN

To assess the surplus value of EMDR compared to waiting list, a single-blind RCT with two groups (EMDR versus waiting list) will be performed. The experimental group will receive ten bi-weekly EMDR sessions of ninety minutes each (with a total duration of 5 weeks). Data will be collected at baseline, 5 weeks (post EMDR-treatment) and three-, six- and twelve-months after onset of the study.
Based on a feasibility analysis, inclusion of patients will take 1 year.
The study will be conducted at Parnassia Psychiatric Institute and GGZ Delfland.

4. STUDY POPULATION

4.1 Population

Participants will be recruited from outpatient clinics of the Parnassia Psychiatric Institute and GGZ Delfland. We aim to include a broad range of patients with PD, because this represents the population that is treated within outpatient, specialized mental health care centers for PD the best.

4.2 Inclusion criteria

In order to be eligible to participate in this study, a subject must meet all of the following criteria:

1. Age eighteen years or older
2. PD classified with the aid of the Structured Clinical Interview for DSM-5 for PD
3. Distress due to traumatic experiences

4.3 Exclusion criteria

A potential subject who meets any of the following criteria will be excluded from participation in this study:

1. Estimated IQ below seventy
2. Inadequate competence in the Dutch language

4.4 Sample size calculation

In order to test the effectiveness of EMDR in reducing the number of PD symptoms over time, relative to waiting list, a linear mixed model (LMM) with intention-to-treat will be used. The RCT of five EMDR sessions revealed an effect size of 0.65 for change in psychological symptoms (Brief Symptom Inventory), 0.62 for psychological functioning (Outcome Questionnaire-45) and 0.56 for change in personality functioning (General Assessment of Personality Disorder) (Hafkemeijer et al., 2020). Since change in personality functioning is also (primarily) assessed in the current study, although with the aid of a different measure (ADP-IV), the effect size for the main analysis is expected to approximate the effect size found by Hafkemeijer et al., 2020. With the aid of standardization by post score standard deviations (standard procedure for meta-analyses) this effect size decreases to 0.46. Therefore, an effect size of 0.43 was estimated to be appropriate for this study. Using Liu and Liang's (1997) equation, assuming a correlation of 0.7 between five repeated measurements, a treatment effect size of 0.43, a power of 0.8, an α significance level of .05, and two treatment conditions, the power analysis

resulted in a total sample size of 132 persons. With an estimated dropout rate of 20% a total of 159 patients is needed. With participation of 6 departments within two institutions our estimation is that it is feasible to include this number of patients within one year.

5. TREATMENT OF SUBJECTS

5.1 Investigational product/treatment

Control condition: Participants in the waiting list condition will be seen once by a study therapist and informed about the course of the study. After three months patients will be offered the opportunity of following a standard treatment for their PD. This period is shorter than the regular waiting time for treatment of PD, which ranges 6 to 12 months.

Experimental condition: The Dutch version of the standard EMDR protocol (de Jongh & ten Broeke, 2019) will be used. The protocol will focus on the most distressing target memory. A total of 10 biweekly sessions of EMDR will be applied with a duration of 90 minutes per session. EMDR therapists are psychologists who have followed at least the basic EMDR training certified according to the guidelines of EMDR Europe. Group supervisions will be provided monthly by consultants in the treatment of EMDR. Additional supervision by telephone or e-mail is available on request. The treatment sessions will be videotaped; 10% will be randomly selected and rated by trained and blinded raters for therapist competence and treatment protocol adherence.

5.2 Use of co-intervention

NA

5.3 Escape medication

NA

6. INVESTIGATIONAL PRODUCT

<This chapter is applicable for research with any product; medicinal product, food product, medical device or other >

NA

6.1 Name and description of investigational product(s)

6.2 Summary of findings from non-clinical studies

<One may refer to the Investigator's Brochure (IB), Investigational Medicinal Product Dossier (IMPD), Summary of Product Characteristics (SPC) or a similar document (if applicable), by mentioning the relevant pages in that document. Be sure that the information is up to date and references to peer reviewed papers in (biomedical/scientific) journals should be given where appropriate.>

6.3 Summary of findings from clinical studies

<See explanatory text of chapter 6.2, including remark>

6.4 Summary of known and potential risks and benefits

<See explanatory text of chapter 6.2, including remark>

6.5 Description and justification of route of administration and dosage

6.6 Dosages, dosage modifications and method of administration

6.7 Preparation and labelling of Investigational Medicinal Product

6.8 Drug accountability

<Please describe the procedures for the shipment, receipt, disposition, return and destruction of the investigational medicinal products.>

7. NON-INVESTIGATIONAL PRODUCT

NA

8. METHODS

8.1 Study parameters/endpoints

8.1.1 Main study parameter/endpoint

- Assessment van DSM Persoonlijkheidsstoornissen-IV (ADP-IV) vragenlijst (α= .76-.77; Doering et al., 2007; Schotte et al., 1998; 2004)
- Clinician-Administered PTSD Scale for DSM-5 (CAPS-5; α= .90; Boeschoten et al., 2014a; 2018)

8.1.2 Secondary study parameters/endpoints (if applicable)

- Structured Clinical Interview for DSM 5 Personality Disorders (SCID-5-PD; *κ*= .6-.83; Maffei et al., 1997)
- Jeugd Trauma Vragenlijst (JTV, Bernstein et al., 1997)
- Life Events Checklist for the DSM-5 (LEC-5, Gray et al., 2004)
- Level of Personality Functioning Scale - Brief Form 2.0 (LPFS-BF 2.0, Hutsebaut et al., 2015)
- PTSD Check List (PCL; α= .93; Boeschoten et al., 2014b; Van Praag, 2020)
- Difficulties in Emotion Regulation Scale (DERS; α= .81; Gratz & Roemer, 2004; Neuman et al., 2010)
- Brief State Paranoia Checklist (BSPC, Schlier, Moritz, & Lincoln, 2016)
- Outcome Questionnaire-45 (OQ-45; α= .93; De Jong et al., 2007; 2008)
- Euro-Quality of Life 5 dimension 5 level version (EQ-5D-5L; α not clear; EuroQol Group, 1990)
- Treatment inventory Cost In Psychiatric Patients (TiC-P; κ= .49-.84; Bouwmans et al., 2013)
- Mental Health Quality of Life questionnaire (MHQoL, Cronbach’s α = .86, Ware, Kosinski, Dewey, & Gandek, 2001)
- A self-designed interview about experiences with EMDR therapy (see: appendix A).
- Number of dropouts

8.1.3 Other study parameters (if applicable)

- Broad demographic data will be collected to explore which patient will (not) benefit from EMDR.

8.2 Randomisation, blinding and treatment allocation

The study is single-blind. Considering the nature of the study, double-blinding is impossible; study participants know that they are receiving EMDR. However, researchers for data collection can be held ignorant of the condition the participant is in. Random allocation to one of the treatment arms will be performed with the use of the website: www.randomization.com. A password protected randomization list that is accessible by the coordinating and principle researchers will be kept on the server. The researchers will establish contact between participant and EMDR therapist.

8.3 Study procedures

1. Patients will be recruited in two ways; 1. by means of posters hanging in the waiting room; patients can announce themselves to their treating therapist/physician or contact the researchers directly 2. Patients with a PD will be informed by the therapist/physician about the study. The therapist/physician informs the researchers, who will send information to the patient by mail and call the patients to invite them for an appointment. Interested patients will receive further information about the study during an appointment with one of the researchers. Furthermore, the researcher will check eligibility for inclusion. At the end of the appointment patients will be given informed consent forms. The patients are allowed to take the time they need to make a decision about participation. Patients who decide to participate will be asked to sign informed consent.
2. Diagnostic instruments before inclusion: a. Any PD diagnosis will be confirmed using the SCID-5-PD
3. Informed consent: During an intake interview with the researchers, patients are informed about the study verbally and formally. If the patient had enough time to consider whether he/she wants to participate into this study, the patient is asked to give written, informed consent.
4. Randomization: After patients are included and have given informed consent, patients are randomized into one of the two conditions.
5. Questionnaires will be administered at baseline (before randomization), after weeks (post EMDR) and three, six and twelve months after onset of the study.
6. Interview: all participants in the EMDR group will be requested to participate in a qualitative interview about their experiences with EMDR therapy.
7. Patients in the experimental group are treated with ten biweekly, ninety minute, EMDR sessions. These patients are compared with a waiting list condition. After three months of follow-up patients can decide whether they want to have standard treatment for PD.

8.4 Withdrawal of individual subjects

Subjects can leave the study at any time for any reason if they wish to do so without any consequences. The investigator can decide to withdraw a subject from the study for urgent medical reasons.

8.4.1 Specific criteria for withdrawal (if applicable)

8.5 Replacement of individual subjects after withdrawal

Inclusion is continued until 159 participants have been included. We attempt to collect reasons for withdrawal. However, patients are not obliged to give a reason.

8.6 Follow-up of subjects withdrawn from treatment

Patients will be asked to continue the assessments if they prematurely stop the treatment sessions. It is attempted to collect reasons for withdrawal. However, patients are not obliged to give a reason for withdrawal and withdrawal of assessment.

8.7 Premature termination of the study

Aside from individual withdrawal, we expect no event that will lead to premature termination of the study. However, if this study will end prematurely, this will have no influence on the treatment of patients for their PD.

9. SAFETY REPORTING

9.1 Temporary halt for reasons of subject safety

In accordance to section 10, subsection 4, of the WMO, the sponsor will suspend the study if there is sufficient ground that continuation of the study will jeopardize subject health or safety. The sponsor will notify the accredited METC without undue delay of a temporary halt including the reason for such an action. The study will be suspended pending a further positive decision by the accredited METC. The investigator will take care that all subjects are kept informed.

9.2 AEs, SAEs and SUSARs

9.2.1 Adverse events (AEs)

Adverse events are defined as any undesirable experience occurring to a subject during the study, whether or not considered related to [the investigational product / trial procedure/ the experimental intervention]. All adverse events reported spontaneously by the subject or observed by the investigator or his staff will be recorded.

9.2.2 Serious adverse events (SAEs)

A serious adverse event is any untoward medical occurrence or effect that

- results in death;
- is life threatening (at the time of the event);
- requires hospitalization or prolongation of existing inpatients' hospitalization;
- results in persistent or significant disability or incapacity;
- is a congenital anomaly or birth defect; or
- any other important medical event that did not result in any of the outcomes listed above due to medical or surgical intervention but could have been based upon appropriate judgement by the investigator.

An elective hospital admission will not be considered as a serious adverse event.

< *Please describe the procedures for handling the serious adverse events. If certain SAEs do not require immediate reporting by the investigator to the sponsor, please specify.*>

The investigator will report all SAEs to the sponsor without undue delay after obtaining knowledge of the events, except for the following SAEs: <*specify which*

SAEs do not require immediate reporting by the investigator to the sponsor, if applicable> .

The sponsor will report the SAEs through the web portal *ToetsingOnline* to the accredited METC that approved the protocol, within 7 days of first knowledge for SAEs that result in death or are life threatening followed by a period of maximum of 8 days to complete the initial preliminary report. All other SAEs will be reported within a period of maximum 15 days after the sponsor has first knowledge of the serious adverse events.

< *If certain SAEs do not require(expedited) reporting to the accredited METC, please specify these SAEs as well as the frequency of reporting of these SAEs in line listings, or in a annual safety report or otherwise.>*

9.2.3 Suspected unexpected serious adverse reactions (SUSARs)

<This c*hapter is only applicable for studies with an investigational medicinal product*>
Adverse reactions are all untoward and unintended responses to an investigational product related to any dose administered.

Unexpected adverse reactions are SUSARs if the following three conditions are met:

1. the event must be serious (see chapter 9.2.2);
2. there must be a certain degree of probability that the event is a harmful and an undesirable reaction to the medicinal product under investigation, regardless of the administered dose;
3. the adverse reaction must be unexpected, that is to say, the nature and severity of the adverse reaction are not in agreement with the product information as recorded in:
   - Summary of Product Characteristics (SPC) for an authorised medicinal product;
   - Investigator's Brochure for an unauthorised medicinal product.

The sponsor will report expedited the following SUSARs through the web portal *ToetsingOnline* to the METC *<reporting via webportalToetsingOnline is only applicable for investigator initiated studies>*:

- SUSARs that have arisen in the clinical trial that was assessed by the METC;

- SUSARs that have arisen in other clinical trials of the same sponsor and with the same medicinal product, and that could have consequences for the safety of the subjects involved in the clinical trial that was assessed by the METC.

The remaining SUSARs are recorded in an overview list (line-listing) that will be submitted once every half year to the METC. This line-listing provides an overview of all SUSARs from the study medicine, accompanied by a brief report highlighting the main points of concern.
The expedited reporting of SUSARs through the web portal Eudravigilance or ToetsingOnline is sufficient as notification to the competent authority.

The sponsor will report expedited all SUSARs to the competent authorities in other Member States, according to the requirements of the Member States.

The expedited reporting will occur not later than 15 days after the sponsor has first knowledge of the adverse reactions. For fatal or life threatening cases the term will be maximal 7 days for a preliminary report with another 8 days for completion of the report.

9.3 Annual safety report

In addition to the expedited reporting of SUSARs, the sponsor will submit, once a year throughout the clinical trial, a safety report to the accredited METC, competent authority, and competent authorities of the concerned Member States.
This safety report consists of:

- a list of all suspected (unexpected or expected) serious adverse reactions, along with an aggregated summary table of all reported serious adverse reactions, ordered by organ system, per study;
- a report concerning the safety of the subjects, consisting of a complete safety analysis and an evaluation of the balance between the efficacy and the harmfulness of the medicine under investigation.

9.4 Follow-up of adverse events

All AEs will be followed until they have abated, or until a stable situation has been reached. Depending on the event, follow up may require additional tests or medical procedures as indicated, and/or referral to the general physician or a medical specialist.

SAEs need to be reported till end of study within the Netherlands, as defined in the protocol

9.5 [Data Safety Monitoring Board (DSMB) / Safety Committee]

EMDR is an evidence-based treatment for PTSD and is found to be safe. A DSMB is therefore not instituted.

10. STATISTICAL ANALYSIS

Most data are quantitative and will be analyzed using SPSS 25 (SPSS Inc., Chicago, IL). The aim is to collect complete data. It is attempted to avoid missing data. In case that missing data occurs, patients with missing data will not be excluded, since multiple measurement points are used.

10.1 Primary study parameter(s)

The primary study parameter is the number of diagnosed PD symptoms, over five moments of measurement. In order to test the effectiveness of EMDR in reducing the number of PD symptoms over time, a linear mixed model (LMM) with intention-to-treat will be used. Within-subjects factor ‘Time’ has five levels (baseline vs. post-intervention vs. three-month vs. six-month vs. twelve-month follow-ups) and between-subjects factor ‘Group’ has two (EMDR vs. waiting list). Corrected post-hoc analyses will be performed where significant effects are found.

10.2 Secondary study parameter(s)

Mixed models with intention-to-treat will be used to compare EMDR and waiting list qua (1) the decline of diagnoses of PD, (2) the severity of psychotrauma and PTSD symptoms and (3) the severity of difficulties in emotion regulation and interpersonal functioning.
The economic evaluation will be undertaken according to the Dutch guidelines for costs effectiveness studies taken a societal perspective (Hakkaart et al., 2016). The severity (i.e., number of symptoms) of PD, aided by the ADP-IV, will be used for effectiveness. The TiC-P is used to collect data on utilization of medical services and productivity loss. Utility scores are estimated using the EQ-5D-5L and MHQoL will measure the effects. A cost-utility analysis and a cost-effectiveness analysis (CEA) are conducted. The cost-utility is calculated as the incremental costs and utilities, which yields a cost per QALY estimate. The cost-effectiveness is expressed in the incremental costs and scores on the SCID-5-PD.
Multilevel modeling is used to model cost-effectiveness. For assessing the uncertainty, cost effectiveness acceptability curves and cost effectiveness planes will be created after bootstrapping. The acceptability curve illustrates the probability that the cost-effectiveness ratio will be accepted for different thresholds. In a cost-effectiveness plane, both incremental costs and incremental effects are plotted to account for combinations. Sensitivity analyses will be performed after the analysis if necessary.
Qualitative data on patients’ experiences with EMDR therapy will be summarised by the researchers and provided to participants for respondent validation. The researchers will

thoroughly read all transcripts, highlight all potentially relevant excerpts and sort them based on the research questions. These sorted data will then be independently coded by the researchers; excerpts will be grouped based on their similarities. Then, these groupings will be identified/themed. Consequently, the researchers will confer and attempt to reach consensus on all groupings and themes, resulting in a preliminary codebook. The codebook and two information rich transcripts as examples will be presented to the study group. Themes will be reviewed, focussing on understanding the data and confirming that the data still correspond to the assigned themes.

10.3 Other study parameters

Sample characteristics will be collected, presented and used in a regression analysis to explore individual predictors of success with EMDR.

10.4 Interim analysis

NA

11. ETHICAL CONSIDERATIONS

11.1 Regulation statement

The present study will be conducted according to the principles of the Declaration of Helsinki (World Medical Association, 2013) and in accordance with the Medical Research Involving Human Subjects Act (WMO).

11.2 Recruitment and consent

Recruitment will be at GGZ Delfland and multiple institutions of Parnassia Psychiatric Institute. Patients will be recruited in two ways. 1. By means of posters hanging in the waiting room; patients can announce themselves to their treating therapist/physician or the investigator. 2. Patients with the relevant diagnosis will be informed by their therapist/physician about the research. If the patient is interested, the therapist/physician informs the researchers, who will call the patients to invite them for an appointment. At this appointment, the researchers will provide the patients with further information and check eligibility for inclusion. If patients meet the inclusion criteria, they will receive the informed consent form, which they are allowed to sign right away, or whenever they feel ready. The written, informed consent of the patient will be collected by the researcher.

11.3 Objection by minors or incapacitated subjects

NA

11.4 Benefits and risks assessment, group relatedness

Patients with PD suffer from low self-esteem, have severe impairments in the regulation of emotions and interpersonal functioning and their quality of life is low. In a recent study, EMDR reduced BPD symptoms (De Jongh et al. 2020). In another study, psychological symptoms, psychological functioning, and personality functioning were positively affected by 5 EMDR sessions in all PDs (Hafkemeijer et al., submitted for publication). Thus, it is expected that patients who receive EMDR, will experience a reduction in PD symptoms and an improvement in psychological and personality functioning. In addition, EMDR is safe and not associated with an increase of self-injurious behaviour, suicidal gestures, suicides or hospital admissions. is applied in addition to treatment as usual for PD with regular meetings. In case emotions or traumatic intrusions increase in the two or three days after a session of EMDR, patients have the opportunity to discuss their symptoms with the EMDR- and PD-therapists. Also, no risks are expected due to the data collection.

The study requires participants in the control group to not receive treatment for three months. If necessary or if the participant no longer wants to participate, he or she is free to terminate participation.
With participation into this study, patients contribute to knowledge about treatment and can therefore contribute to the quality of treatment.

11.5 Compensation for injury

The sponsor/investigator has a liability insurance which is in accordance with article 7 of the WMO.

The sponsor also has an insurance which is in accordance with the legal requirements in the Netherlands (Article 7 WMO). This insurance provides cover for damage to research subjects through injury or death caused by the study.
The insurance applies to the damage that becomes apparent during the study or within 4 years after the end of the study.

11.6 Incentives (if applicable)

Per data collection (5x), participants receive €25,-, refund of travel expenses and free lunch in the cafeteria.

12. ADMINISTRATIVE ASPECTS, MONITORING AND PUBLICATION

12.1 Handling and storage of data and documents

All patient data will be coded with a participant number. The key to the code will be only managed by the main researchers, Simon Hofman and Karin Slotema, and saved in a locked file with a password that only the main researchers know. On the filled-out forms and in the data files there will be no name, birthdate or identification number. All data will be collected on a computer and transferred into data files. Data files will be saved on the K-drive that only researchers can access. Furthermore, the files will be given a password to ensure only researchers involved in the study can access the files. The handling of the data complies with the Dutch Personal Data Protection Act.

12.2 Monitoring and Quality Assurance

Data will be processed in a data management system (Research manager), which facilitates monitoring. Via this system an Audit-trial will be created. After approval of the METC for this study a monitor will be assigned to monitor this study. The monitor will monitor the study via the research manager data management system. Furthermore, the monitor will visit the site with a minimum frequency of 1 visit per year. After the end of the study a close-out visit will be planned. The monitor will check whether study procedures are followed correctly, and will check the study site's documentation, the participants' source data, eCRF entries, and the correct maintenance of the Investigator Site File.
At the site monthly research meetings take place within the department. In these meetings the project will be discussed with fellow researchers.

12.3 Amendments

Amendments are changes made to the research after a favourable opinion by the accredited METC has been given. All amendments will be notified to the METC that gave a favourable opinion.

All substantial amendments will be notified to the METC and to the competent authority.

Non-substantial amendments will not be notified to the accredited METC and the competent authority, but will be recorded and filed by the sponsor.

12.4 Annual progress report

The sponsor/investigator will submit a summary of the progress of the trial to the accredited METC once a year. Information will be provided on the date of inclusion of the first subject, numbers of subjects included and numbers of subjects that have completed the trial, serious adverse events/ serious adverse reactions, other problems, and amendments.

12.5 Temporary halt and (prematurely) end of study report

The investigator/sponsor will notify the accredited METC of the end of the study within a period of 8 weeks. The end of the study is defined as the last patient's last visit.

The sponsor will notify the METC immediately of a temporary halt of the study, including the reason of such an action.

In case the study is ended prematurely, the sponsor will notify the accredited METC within 15 days, including the reasons for the premature termination.

Within one year after the end of the study, the investigator/sponsor will submit a final study report with the results of the study, including any publications/abstracts of the study, to the accredited METC.

12.6 Public disclosure and publication policy

The results of this study will be published in international peer-reviewed journals and within presentations on national and international conferences.

13. STRUCTURED RISK ANALYSIS

NA

13.1 Potential issues of concern

< In this final paragraph of the research protocol a structured risk analysis which consists of a number of steps is required. The analysis should result in a comprehensive overall synthesis of the direct risks for the research subjects in this study in chapter 13.2. The risk considerations on the various issues listed below should be supported by up to date information and should be clearly described to allow a thorough review by the METC. For details one may refer to the previous chapters, the Investigator's Brochure (IB) or a similar document (if applicable), peer reviewed papers in (biomedical/scientific) journals. The issues below are provided to structure your considerations and allows an efficient communication with the METC when questions arise as a result of the review of your research protocol. The remarks per item are provided as a guidance for describing your considerations. Should issues not be applicable, please indicate so.
For registered products to be used within the indication and ***not*** *in combination with other products chapter 13.1 can be skipped; explain in chapter 13.2 why 13.1 is skipped >*

a. Level of knowledge about mechanism of action

b. Previous exposure of human beings with the test product(s) and/or products with a similar biological mechanism

c. Can the primary or secondary mechanism be induced in animals and/or in *ex-vivo* human cell material?

d. Selectivity of the mechanism to target tissue in animals and/or human beings

e. Analysis of potential effect

f. Pharmacokinetic considerations

g. Study population

h. Interaction with other products

i. Predictability of effect

j. Can effects be managed?

13.2 Synthesis

<should include uncertainties and the unknown and the overall risk:
Make clear what measures have been taken to reduce what risks
Make clear why in your opinion the remaining risks are acceptable for the subjects participating in the study>

14. REFERENCES

Battle CL, Shea MT, Johnson DM, Yen S, Zlotnick C, Zanarini MC, et al. (2004). Childhood maltreatment associated with adult personality disorders: findings from the Collaborative Longitudinal Personality Disorders Study. Journal of Personality Disorders, 18, 193-211.

Bernstein D.P, Ahluvalia T., Pogge D., Handelsman L. (1997), Validity of the Childhood Trauma Questionnaire in an adolescent psychiatric population, Journal of the American Academy of Child & Adolescent Psychiatry, 36, 340-348.

Boeschoten, M.A., Bakker, A., Jongedijk, R.A. & Olff, M. (2014b). PTSD Checklist for DSM-5– Nederlandstalige versie. Uitgave: Stichting Centrum ’45, Arq Psychotrauma Expert Groep, Diemen.

Boeschoten, M.A., Bakker, A., Jongedijk, R.A., van Minnen, A., Elzinga, B.M., Rademaker, A.R. & Olff, M. (2014a). Clinician Administered PTSD Scale for DSM-5 – Nederlandstalige versie. Uitgave: Stichting Centrum ’45, Arq Psychotrauma Expert Groep, Diemen.

Boeschoten, M. A., Van der Aa, N., Bakker, A., Ter Heide, F. J. J., Hoofwijk, M. C., Jongedijk, R. A., ... & Olff, M. (2018). Development and evaluation of the Dutch clinician-administered PTSD scale for DSM-5 (CAPS-5). European Journal of Psychotraumatology, 9, 1546085. https://doi.org/10.1080/20008198.2018.1546085.

Bohus, M., Dyer, A. S., Priebe, K., Krüger, A., Kleindienst, N., Schmahl, C., ... & Steil, R. (2013). Dialectical behaviour therapy for post-traumatic stress disorder after childhood sexual abuse in patients with and without borderline personality disorder: A randomised controlled trial. Psychotherapy and psychosomatics, 82(4), 221-233.

Bouwmans, C., De Jong, K., Timman, R., Zijlstra-Vlasveld, M., Van der Feltz-Cornelis, C., Tan, S. S., & Hakkaart-van Roijen, L. (2013). Feasibility, reliability and validity of a questionnaire on healthcare consumption and productivity loss in patients with a psychiatric disorder (TiC-P). BMC health services research, 13(1), 217. https://doi.org/10.1186/1472-6963-13-217.

De Bont, P. A. J. M., Van Den Berg, D. P. G., Van Der Vleugel, B. M., de Roos, C. J. A. M., De Jongh, A., Van Der Gaag, M., & Van Minnen, A. M. (2016). Prolonged exposure and EMDR for PTSD v. a PTSD waiting-list condition: effects on symptoms of psychosis,

depression and social functioning in patients with chronic psychotic disorders. Psychological medicine, 46(11), 2411-2421.

De Jongh A, Amann BL, Hofmann A, Farrell D, Lee CW. The Status of EMDR Therapy in the Treatment of PTSD 30 Years after its Introduction. Journal of EMDR Practice and Research, 13, 261-269.

De Jong, K., Nugter, M. A., Polak, M. G., Wagenborg, J. E., Spinhoven, P., & Heiser, W. J. (2007). The Outcome Questionnaire (OQ-45) in a Dutch population: A cross-cultural validation. Clinical Psychology & Psychotherapy: An International Journal of Theory & Practice, 14(4), 288-301. https://doi.org/10.1002/cpp.529.

De Jongh, A., Groenland, G. N., Sanches, S,, Bongaerts, H., Voorendonk, E. M., & van Minnen, A. (2020). The impact of brief intensive trauma-focused treatment for PTSD on symptoms of BPD. European Journal of Psychotraumatoly, 14, 11:1721142. doi: 10.1080/20008198.2020.1721142.

De Jongh A., ten Broeke E. (2019). Handboek EMDR. Een geprotocolleerde behandelmethode voor de gevolgen van psychotrauma. Seventh edition, Pearson Benelux. EuroQol Group, The (1990). EuroQol-a new facility for the measurement of health-related quality of life. Health policy, 16(3), 199-208. https://doi.org/10.1016/0168-8510(90)90421-9.

Doering, S., Renn, D., Hofer, S., Rumpold, G., Smrekar, U., Janecke, N., ... & Schussler, G. (2007). Validation of the "assessment of DSM-IV personality disorders (ADP-IV)" questionnaire. *Z Psychosom Med Psychother*, *53*(2), 111-28.

Giesen-Bloo, J., van Dyck, R., Spinhoven, P., van Tilburg, W., Dirksen, C., van Asselt, T., Kremers, I., Nadort, M., & Arntz, A. (2006). Outpatient psychotherapy for borderline personality disorder: randomized trial of schema-focused therapy vs transference-focused psychotherapy. Archives of General Psychiatry, 63, 649-58. doi: 10.1001/archpsyc.63.6.649.

Goldstein, R. B., Chou, S. P., Saha, T. D., Smith, S. M., Jung, J., Zhang, H., Pickering, R. P, Ruan, W. J., Huang, B., & Grant, B. F. (2017). The epidemiology of antisocial behavioral syndromes in adulthood: Results from the national epidemiologic survey on alcohol and related conditions-III. The Journal of Clinical Psychiatry, 78, 90–98. https://doi.org/10.4088/JCP.15m10358.

Gratz, K.L., & Roemer, L. (2004). Multidimensional assessment of emotion regulation and dysregulation: Development, factor structure, and initial validation of the difficulties in emotion regulation scale. Journal of Psychopathology and Behavioral Assessment, 26, 41-54. https://doi.org/10.1023/B:JOBA.0000007455.08539.94.

Gray MJ, Litz BT, Hsu JL, & Lombardo TW. (2004). Psychometric properties of the life events checklist. Assessment, 11, 330–41. https://doi.org/10.1177/1073191104269954.

Hafkemeijer, L. C. S., de Jongh, A., van der Palen, J., & Starrenburg, A. H. A randomized controlled trial of eye movement desensitization and reprocessing (EMDR) therapy versus a waiting list control condition in patients with a personality disorder (submitted).

Hakkaart-van Roijen, L., van der Linden, N., Bouwmans, C., Kanters, T., & Tan, S. S. (2016). Kostenhandleiding: Methodologie van kostenonderzoek en referentieprijzen voor economische evaluaties in de gezondheidszorg. Institute for Medical Technology Assessment, Erasmus University Rotterdam, the Netherlands.

Harned, M. S., Korslund, K. E., & Linehan, M. M. (2014). A pilot randomized controlled trial of Dialectical Behavior Therapy with and without the Dialectical Behavior Therapy Prolonged Exposure protocol for suicidal and self-injuring women with borderline personality disorder and PTSD. Behaviour research and therapy, 55, 7-17.

Hutsebaut, J., Feenstra, D. J., & Kamphuis, J. H. (2015). Development and Preliminary Psychometric Evaluation of a Brief Self-Report Questionnaire for the Assessment of the DSM–5 Level of Personality Functioning Scale: The LPFS Brief Form (LPFS-BF). Personality Disorders: Theory, Research, and Treatment. http://dx.doi.org/10.1037/per0000159

Kredlow, M. A., Szuhany, K. L., Lo, S., Xie, H., Gottlieb, J. D., Rosenberg, S. D., & Mueser, K. T. (2017). Cognitive behavioral therapy for posttraumatic stress disorder in individuals with severe mental illness and borderline personality disorder. Psychiatry research, 249, 86-93.

Liu, G., & Liang, K. Y. (1997). Sample size calculations for studies with correlated observations. *Biometrics*, 937-947. https://doi.org/10.2307/2533554.

Maffei, C., Fossati, A., Agostoni, I., Barraco, A., Bagnato, M., Deborah, D., Namia, C., Novella, L., & Petrachi, M. (1997). Interrater reliability and internal consistency of the

structured clinical interview for DSM-IV axis II personality disorders (SCID-II), version 2.0. J of Pers Dis, 11, 279-284.

Markowitz, J. C., Petkova, E., Neria, Y., Van Meter, P. E., Zhao, Y., Hembree, E., ... & Marshall, R. D. (2015). Is exposure necessary? A randomized clinical trial of interpersonal psychotherapy for PTSD. American Journal of Psychiatry, 172(5), 430-440.

Mavranezouli I, Megnin-Viggars O, Grey N, Bhutani G, Leach J, Daly C, Dias S, et al. Cost-effectiveness of psychological treatments for post-traumatic stress disorder in adults. PLoS One. 2020:15:e0232245. doi: 10.1371/journal.pone.0232245.

McGuire, T. M., Lee, C. W., & Drummond, P. D. (2014). Potential of eye movement desensitization and reprocessing therapy in the treatment of post-traumatic stress disorder. Psychology research and behavior management, 7, 273.

Neumann, A., van Lier, P.A.C., Gratz, K.L., & Koot, H.M. (2010). Multidimensional assessment of emotion regulation difficulties in adolescents using the Difficulties in Emotion Regulation Scale. Assessment, 17, 138-149. https://doi.org/10.1177%2F1073191109349579.

Newton-Howes, G., Tyrer, P., Anagnostakis, K., Cooper, S., Bowden-Jones, O., & Weaver, T. (2010). The prevalence of personality disorder, its comorbidity with mental state disorders, and its clinical significance in community mental health teams. Social psychiatry and psychiatric epidemiology, 45(4), 453-460.

Niemantsverdriet, M. B. A., Slotema, C. W., Blom, J. D., Franken, I.H., Hoek, H. W., Sommer, I. E. C., & van der Gaag, M. (2017). Hallucinations in borderline personality disorder: prevalence, characteristics and associations with comorbid symptoms and disorders. Scientific Reports, 7, 13920. doi: 10.1038/s41598-017-13108-6.

Schotte, C. K., Doncker, D. A. D., Dmitruk, D., Mulders, I. V., D'Haenen, H., & Cosyns, P. (2004). The ADP-IV questionnaire: differential validity and concordance with the semi-structured interview. *Journal of Personality Disorders*, *18*(4), 405-419.

Schotte, C. K. W., De Doncker, D., Vankerckhoven, C., Vertommen, H., & Cosyns, P. (1998). Self-report assessment of the DSM-IV personality disorders. Measurement of trait and distress characteristics: the ADP-IV. *Psychological medicine*, *28*(5), 1179-1188.

Seo, J., & Choi, J. Y. (2018). Social defeat as a mediator of the relationship between childhood trauma and paranoid ideation. Psychiatry Research, 260, 48-52.

Schlier B, Moritz S, Lincoln TM. (2016). Measuring fluctuations in paranoia: Validity and psychometric properties of brief state versions of the Paranoia Checklist. Psychiatry Research, 241, 323-32. doi: 10.1016/j.psychres.2016.05.002.

Sheehan L, Nieweglowski K, & Corrigan P. The Stigma of Personality Disorders. Curr Psychiatry Rep. 2016;18:11. doi: 10.1007/s11920-015-0654-1.

Slotema, C. W., Van den Berg, D. P., Driessen, A., Wilhelmus, B., & Franken, I. H. (2019). Feasibility of EMDR for posttraumatic stress disorder in patients with personality disorders: a pilot study. European journal of psychotraumatology, 10(1), 1614822.

Slotema CW, Blom JD, Niemantsverdriet MB, Deen M, Sommer IE. Comorbid diagnosis of psychotic disorders in BPD: prevalence and influence on outcome. Front Psychiatry 2018;9:84, doi:10.3389/fpsyt.2018.00084.

Slotema, C. W., Wilhelmus, B., Arends, L. R., & Franken, I. H. (2020). Psychotherapy for posttraumatic stress disorder in patients with borderline personality disorder: a systematic review and meta-analysis of its efficacy and safety. *European Journal of psychotraumatology*, *11*(1), 1796188.

Svartberg, M., Stiles, T. C., & Seltzer, M. H. (2004). Randomized, controlled trial of the effectiveness of short-term dynamic psychotherapy and cognitive therapy for cluster C personality disorders. American Journal of Psychiatry, 161, 810-7. doi: 10.1176/appi.ajp.161.5.810.

Tyrer, P., Reed, G. M., & Crawford, M. J. (2015). Classification, assessment, prevalence, and effect of personality disorder. The Lancet, 385(9969), 717-726.

Van Emmerik, A. A. P., Schoorl, M., Emmelkamp, P. M. G., & Kamphuis, J. H. (2006). Psychometric evaluation of the Dutch version of the posttraumatic cognitions inventory (PTCI). Behaviour Research and Therapy, 44(7), 1053-1065. https://doi.org/10.1016/j.brat.2005.07.002.

Van Praag, D. L., Fardzadeh, H. E., Covic, A., Maas, A. I., & von Steinbüchel, N. (2020). Preliminary validation of the Dutch version of the Posttraumatic stress disorder checklist for DSM-5 (PCL-5) after traumatic brain injury in a civilian population. PloS one, 15(4), e0231857. https://doi.org/10.1371/journal.pone.0231857.

Voestermans, D., Eikelenboom, M., Rullmann, J., Wolters-Geerdink, M., Draijer, N., Smit, J. H., ... & van Marle, H. J. (2020). The Association Between Childhood Trauma and Attachment Functioning in Patients With Personality Disorders. Journal of Personality Disorders, 1-19.

Ware, J. E., Kosinski, M., Dewey, J. E., & Gandek, B. (2001). A manual for users of the SF-8 health survey. Lincoln, RI: QualityMetric.

Yen, S., Shea, M. T., Battle, C. L., Johnson, D. M., Zlotnick, C., Dolan-Sewell, R., ... & Zanarini, M. C. (2002). Traumatic exposure and posttraumatic stress disorder in borderline, schizotypal, avoidant, and obsessive-compulsive personality disorders: fingings from the collaborative longitudinal personality disorders study. The Journal of nervous and mental disease, 190(8), 510-518.

Zanarini, M. C. (2000). Childhood experiences associated with the development of borderline personality disorder. Psychiatric Clinics of North America, 23(1), 89-101.

Zanarini, M. C., Frankenburg, F. R., Dubo, E. D., Sickel, A. E., Trikha, A., Levin, A., & Reynolds, V. (1998). Axis I comorbidity of borderline personality disorder. American Journal of psychiatry, 155(12), 1733-1739.

Zanarini, M. C., Gunderson, J. G., Marino, M. F, Schwartz, E. O., & Frankenburg, F. R. (1989). Childhood experiences of borderline patients. Comprehensive Psychiatry, 1, 18-25. doi: 10.1016/0010-440x(89)90114-4.

Appendix A. Interview experiences with EMDR.

VOOR DE ONDERZOEKER*: We willen inzicht krijgen in de ervaringen van patiënten met EMDR-therapie. De volgende (deels overlappende) vragen kunnen daarbij gesteld worden. De onderzoeker kan vervolgens doorvragen om additionele informatie te vergaren. Het einddoel is om duidelijk te maken hoe de patiënt zijn of haar behandeling met EMDR heeft ervaren.*

1. Hoe kijkt u terug op uw EMDR-behandeling?
2. Vindt u dat uw EMDR-behandeling succesvol is geweest? Waar merkt u dat aan? Welke specifieke klachten zijn verminder of verdwenen?
3. In hoeverre verschilt uw leven van voor en van na de EMDR-behandeling?
4. Had de EMDR-behandeling voor u ook nadelen? Wat was concreet het nadeel?
5. Specifiek, in hoeverre merkte u bijeffecten van de EMDR kort na een sessie?
6. Vond u de voordelen van de behandeling opwegen tegen de nadelen?
7. Heeft de EMDR-behandeling ook een (positief of negatief) effect gehad op uw andere behandelingen? Heeft u ervaringen uit de EMDR-behandeling nader besproken in uw hoofdbehandeling?
8. Wat vond u van het moment waarop u EMDR-behandeling heeft gevolgd? Zou EMDR eerder of later in het proces moeten worden gegeven?
9. Wat vond u van uw EMDR-behandelaar? Wat hielp u en wat niet?
10. Hoe zou u, op een schaal van 1 (verschrikkelijk) tot 10 (uitstekend), de therapeutische band met uw behandelaar beoordelen?
11. Bent u tevreden over de keuze om destijds een EMDR-behandeling te gaan starten? Zou u dezelfde keuze nog eens maken?
12. Zou u het anderen (met dezelfde klachten) aanraden ook een EMDR-behandeling te volgen? Waarom?
13. Heeft u nog verbeteringen voor onze EMDR-behandeling?
