# Supplementary material for: Eye Movement Desensitization and Reprocessing Therapy in Persons With Personality Disorders: A Randomized Clinical Trial
Source: JAMA Netw Open. 2025 Sep 25;8(9):e2533421. doi: 10.1001/jamanetworkopen.2025.33421 (PMC12464786; doi:10.1001/jamanetworkopen.2025.33421)
Supplement: Supplement 2. — Data Sharing Statement [file jamanetwopen-e2533421-s002.pdf]

Data Sharing Statement

Hofman. Effectiveness of Eye Movement Desensitization and Reprocessing Therapy on Personality Disorders. *JAMA Netw Open*. Published September 25, 2025. doi:10.1001/jamanetworkopen.2025.33421

Data
Additional Information: Netherlands Trial Register: NL9078
Data available: No

Additional Information
Explanation for why data not available: Access to the data will be considered upon reasonable request to the corresponding author.
